# Phase-Locked Signals Elucidate Circuit Architecture of an Oscillatory Pathway

**DOI:** 10.1371/journal.pcbi.1001040

**Published:** 2010-12-23

**Authors:** Andreja Jovic, Bryan Howell, Michelle Cote, Susan M. Wade, Khamir Mehta, Atsushi Miyawaki, Richard R. Neubig, Jennifer J. Linderman, Shuichi Takayama

**Affiliations:** 1 Biomedical Engineering Department, University of Michigan, Ann Arbor, Michigan, United States of America; 2 Pharmacology Department, University of Michigan, Ann Arbor, Michigan, United States of America; 3 Department of Chemical Engineering, University of Michigan, Ann Arbor, Michigan, United States of America; 4 Laboratory for Cell Function and Dynamics, Advanced Technology Development Center, Brain Science Institute, Wako City, Saitama, Japan; 5 Macromolecular Science and Engineering Department, University of Michigan, Ann Arbor, Michigan, United States of America; University of Massachusetts at Amherst, United States of America

## Abstract

This paper introduces the concept of phase-locking analysis of oscillatory cellular signaling systems to elucidate biochemical circuit architecture. Phase-locking is a physical phenomenon that refers to a response mode in which system output is synchronized to a periodic stimulus; in some instances, the number of responses can be fewer than the number of inputs, indicative of skipped beats. While the observation of phase-locking alone is largely independent of detailed mechanism, we find that the properties of phase-locking are useful for discriminating circuit architectures because they reflect not only the activation but also the recovery characteristics of biochemical circuits. Here, this principle is demonstrated for analysis of a G-protein coupled receptor system, the M3 muscarinic receptor-calcium signaling pathway, using microfluidic-mediated periodic chemical stimulation of the M3 receptor with carbachol and real-time imaging of resulting calcium transients. Using this approach we uncovered the potential importance of basal IP3 production, a finding that has important implications on calcium response fidelity to periodic stimulation. Based upon our analysis, we also negated the notion that the Gq-PLC interaction is switch-like, which has a strong influence upon how extracellular signals are filtered and interpreted downstream. Phase-locking analysis is a new and useful tool for model revision and mechanism elucidation; the method complements conventional genetic and chemical tools for analysis of cellular signaling circuitry and should be broadly applicable to other oscillatory pathways.

## Author Summary

Key to robust discernment of cell circuit architecture is to have as many distinct response features as possible for comparison and evaluation. One under-appreciated characteristic of oscillatory circuits is that under periodic stimulation, these systems will exhibit responses synchronized to this stimulatory input, a phenomenon termed phase-locking. We demonstrate that phase-locked response characteristics vary noticeably depending on circuit activation and recovery properties; these response characteristics thereby provide a unique set of criteria for oscillatory circuit architecture analysis. The concept is validated through experiments on an oscillatory calcium pathway in mammalian cells; the experimental setup allowed us to explore, for the first time, the properties of chemically induced phase-locking of intracellular signals. Observations of this phenomenon were then used to test the predictions of several existing mathematical models of calcium signaling. Most of the models we evaluated were unable to match all our experimental observations, suggesting that current models are missing mechanistic elements in the context of calcium signaling for the cell type and receptor/stimulant tested. The observations of phase-locking further led us to identify one simple mechanistic modification that would account for all the experimental observations. The techniques and methodology presented should be broadly applicable to a variety of biological oscillators.

## Introduction

Determining the circuit architecture of cellular signaling pathways is challenging. Analysis using perturbative tools including siRNA [1], [2], protein over-expression [3], chemical inhibitors [4], or caged compounds [5] usually reveal multiple plausible models that require further refinements and clarification, not just one definitive conclusion. Thus, there is always a need for additional tests and readouts that shed light on signaling circuit architecture in a robustly discriminating manner.

Most perturbations applied to biochemical circuit analysis are genetic or chemical in nature and alters the circuit architecture itself. Furthermore, the analysis usually looks at how such perturbations change signaling in response to a single step change with no further time variation in stimulation parameters. While these types of analyses are very useful, the circuit-destructive and temporally non-varying nature limits information that can be obtained concerning dynamic properties of the intact signaling system [6]. We hypothesized that analysis of the frequency-dependent response characteristics of the intact biological oscillator circuit to periodic extracellular chemical stimulation would reveal critical activation and recovery properties of biological oscillators to enable elucidation of molecular mechanisms. Here we demonstrate and validate this concept for the oscillatory calcium pathway of the G-protein coupled receptor (GPCR) M3 muscarinic system.

The biochemical recovery properties of this system were evaluated by reducing the rest period between pulses of the M3 ligand, carbachol (CCh), and observing the resulting calcium responses. We noted the emergence of beat skipping upon periodic stimulation. The phenomenon whereby an oscillatory system becomes synchronized to a periodic stimulation input is referred to as phase-locking. As the rest period between stimulation pulses was decreased, the number of system responses of the signaling pathway of interest became less than the number of stimulatory inputs thereby revealing biochemical pathway recovery properties not attainable by continuous stimulation. Furthermore, the skipped beats often were not completely absent, but instead appeared as small calcium transients that we here termed “sub-threshold” spikes; these have been observed previously in electrical responses of cellular systems [7]. The sub-threshold spikes provided insight into the activation properties of the signaling system. The complete absence of a sub-threshold spike would suggest that a switch-like mechanism produced calcium spikes; their presence, however, would suggest that a graded mechanism was more plausible.

These experimental observations of phase-locking properties were compared to the activation and recovery properties of nine models of oscillatory calcium signaling; while these models exclusively deal with the temporal dynamics of calcium signaling and we note that more elaborate models that also include spatial dynamics and IP3 receptor noise are available [8], [9]. In the main text we focus upon two highly different models: the Chay et al. model [10], and the positive feedback Politi et al. model [11]. The former model is the first that theoretically analyzed calcium dynamics in chemically-induced phase-locking; the latter model was recently published, features experimental work to support its proposed mechanisms, and carries dynamic features from previous models and experiments [12], [13], [14]. In addition, both models are able to account for a wide range of calcium oscillation periods (10s of seconds to minutes) upon continuous stimulation. The activation properties of the Chay et al. model are characterized by switch-like activation of phospholipase C (PLC) by G-protein, and it also features basal inositol triphosphate (IP3) production, which represents a recovery mechanism that ensures that IP3 quickly returns to its pre-stimulus levels. The Politi et al. model does not have such a recovery mechanism, and features graded PLC activation. To produce oscillations in the Chay et al. model, the products of the switch-like activation of PLC (IP3 and diacylglycerol) negatively feedback on upstream pathway components (G-proteins). In the Politi et al. model, IP3, produced by graded activation of PLC, feeds back on downstream elements (IP3 receptor) and calcium feeds back upon upstream elements (PLC) to create oscillations. A large number of oscillatory calcium models feature the aforementioned feedback mechanisms [15], [16], [17], [18], [19].

Under continuous stimulation, both models exhibit calcium oscillations with increasing frequencies upon increasing stimulation concentration, as seen in a host of experimental data [20], [21], [22]. Both models were thus appropriate but indistinguishable by conventional stimulation methods. The discriminating features provided by phase-locking analysis, however, revealed that neither of the calcium models correctly predicted all the experimental behaviors based upon their activation and recovery dynamics. Furthermore, by analyzing the sources of discrepancy between the predictions and experiments, we were able to propose a mechanism and parameter modification to account for all the experimental observations of phase-locking.

Although phase-locking can be thought of as a general property of biological oscillators [23], it has not been previously explored experimentally in the context of chemical stimulations. While recent reports have claimed that phase-locking events are largely independent of detailed mechanism [24], we show that the properties of phase-locking can be employed for elucidation of some of the activation and recovery properties of an oscillatory calcium system. We demonstrate that phase-locking, which can only be observed using temporally patterned stimulation, complements conventional chemical and genetic tools for elucidating non-linear oscillatory pathways.

## Results/Discussion

**Figure 1:**
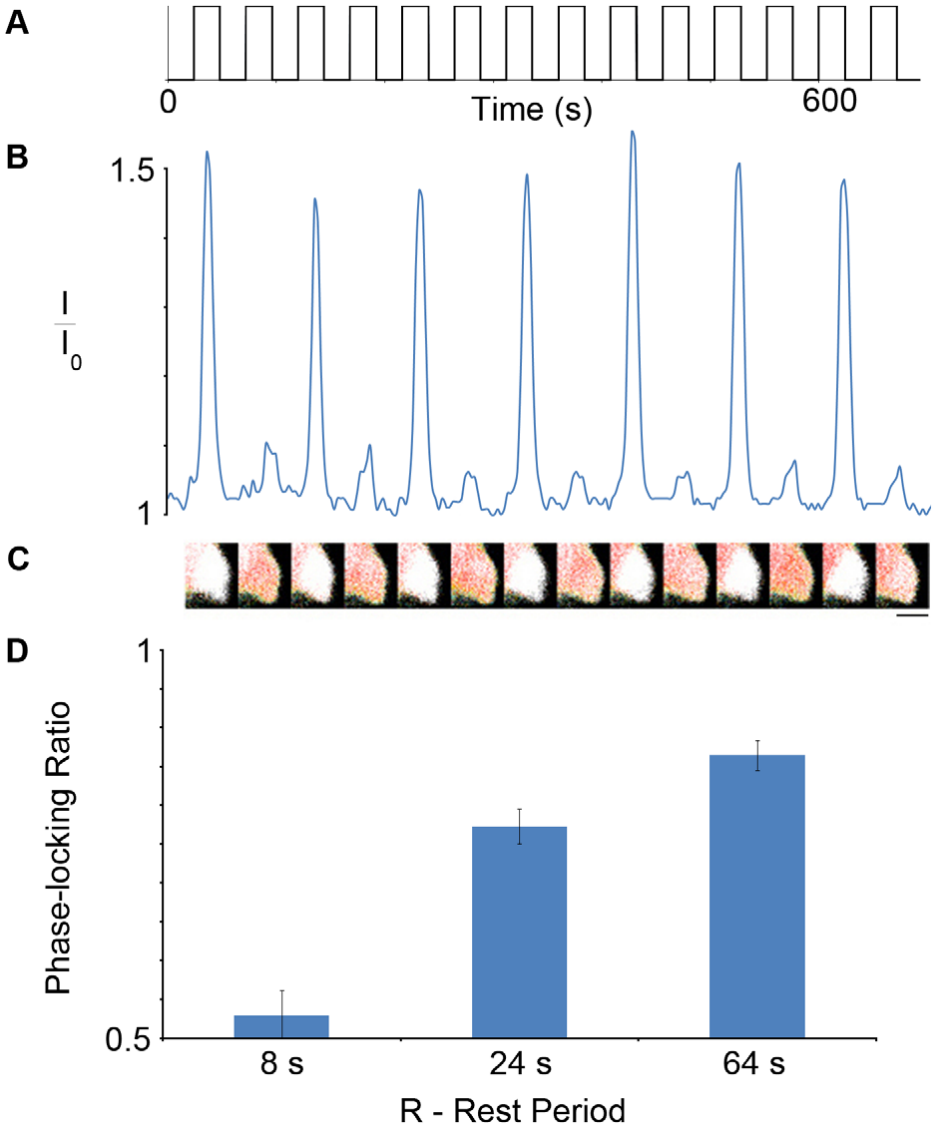
Individual HEK293 cell exhibiting a calcium phase-locking ratio of 0.5 upon square-wave carbachol (CCh) stimulation. a) Temporal pattern of CCh stimulation; the cell was addressed with 25 nM CCh for 24 s, followed by a rest period of 24 s. b) Phase-locked calcium response monitored by normalized FRET ratio (I/I_0_); I_0_ is the minimum FRET ratio obtained during an experimental run to which the remaining ratios (I) were normalized. c) FRET images of the cellular calcium responses. (scale bar = 10 microns) d) Effect of rest period (R) on average phase-locking ratio; cells were exposed to three different rest period values, while the stimulant concentration (C) was fixed at 10 nM CCh and stimulation duration (D) was fixed at 24 s. Bars indicate the S.E.M., representative of three experiments for each experimental condition; for each experiment, the responses of least 20 cells were recorded, resulting in totals between 85 and 106 cells for each experimental condition. All pairs of experimental conditions were statistically significant as determined by the unpaired Student t-test (p<0.05).

We assessed cellular responses to square-wave stimulation through use of a microfluidic platform [modification of 25], which enabled exploration of phase-locked rhythms induced by chemical input signals (Fig. 1a–c). With fixed stimulant concentration (C) and stimulation duration (D), increases in the rest period (R) resulted in increases in the phase-locking ratio (Fig. 1d); phase-locking ratios were calculated by dividing the number of system responses by the number of chemical inputs (See Fig. 1,2 in Text S1). Analysis of the phase-locking rhythms also uncovered the existence of sub-threshold calcium spikes in individual cellular calcium responses (Fig. 1b). In addition, we explored the phase-locking trends induced by varying C and D (See Fig. 3a, b in Text S1). These observations collectively provided robust discrimination markers for rigorous evaluation of mathematical models of oscillatory calcium signaling in order to elucidate molecular mechanisms.

**Figure 2:**
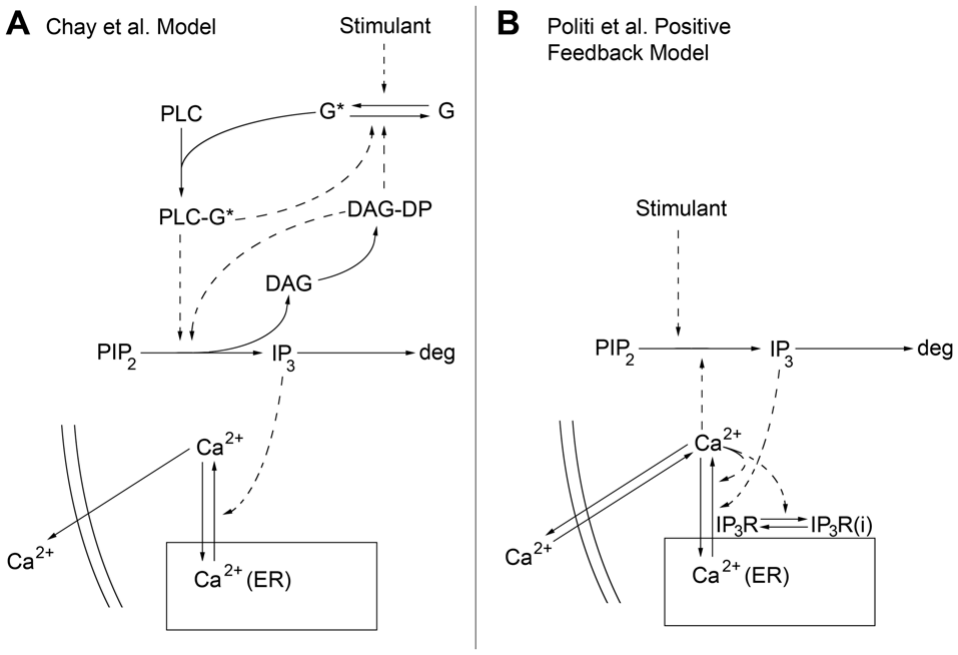
Mathematical model schematics. a) Mathematical model developed by Chay et al. [10] b) Mathematical model developed by Politi et al. [11]. Dashed arrows indicate positive feedback. DAG = diacylglycerol; DAG-DP = DAG-dependent protein; IP3R = IP3 Receptor; IP3R(i) = inactivated IP3R; Ca2+(ER) = Endoplasmic Reticulum calcium.

**Figure 3:**
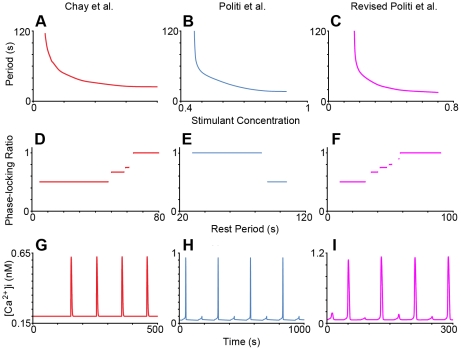
Phase-locking analysis of oscillatory calcium models. Behaviors of the Chay et al. model (left column), the Politi et. al. model (middle column), and revised Politi et al. model (basal IP3 production = 0.3 µM/s) (right column) under continuous are depicted in a–c, and behaviors under periodic stimulation are depicted in d–i. a)–c) Oscillation period vs. continuous stimulant concentration for all three models; the shapes of these graphs are highly similar and thus cannot be used to distinguish between the different mechanisms. In a) stimulant concentration has units of 1/s and represents the rate of receptor-mediated G-protein activation. For b) and c) the stimulant concentrations have units of µM/s and represent the maximal rates of IP3 production. d) Phase-locking ratio vs. Rest Period (R) (Concentration (C) = 0.03 1/s, Stimulation Duration (D) = 10 s); as the rest period between stimulation events is increased for the Chay et al. model, the phase-locking ratio increased, as was observed experimentally in Fig. 1d. e) Phase-locking ratio vs. R (C = 0.8 µM/s D = 30 s); for the Politi et al. model, the phase-locking ratio decreases as the rest period is increased, opposite of experimental results. f) Phase-locking ratio vs. R (C = 0.3 µM/s D = 10 s); the revised Politi et al. model exhibits recovery properties consistent with experimental results. Graphs g–i depict individual intracellular calcium vs. time graphs, where the respective models were periodically stimulated with the given values for stimulation parameters C, D, and R; periodic stimulation of these systems revealed the presence or absence of sub-threshold spikes, as observed in Fig. 1b. g) Intracellular calcium vs. time, C = 0.03 1/s, D = 10 s, R = 40 s. h) Intracellular calcium vs. time, C = 0.8 µM/s, D = 30 s, R = 95 s. i) Intracellular calcium concentration vs. time, with C = 0.3 µM/s, D = 10 s, R = 30 s.

Nine oscillatory calcium models were chosen as a test set against our experimental results, based upon the inability to discriminate their behaviors using continuous stimulation despite significant differences in their activation and recovery mechanisms. Here we show phase-locking analysis of two of these models: the Chay et al. model [10] and the Politi et al. model [11] (Fig. 2). Under continuous stimulation, both the Chay et al. and Politi et al. models exhibited oscillatory calcium responses in physiologically relevant frequency ranges (Fig. 3a–c); furthermore, both depicted the same behavior as the strength of stimulation was increased, as depicted in Fig. 3a and b. We demonstrate that phase-locking analysis is able to effectively dissect the differences in recovery and activation properties between the models (Fig. 3d–i).

**Figure 4:**
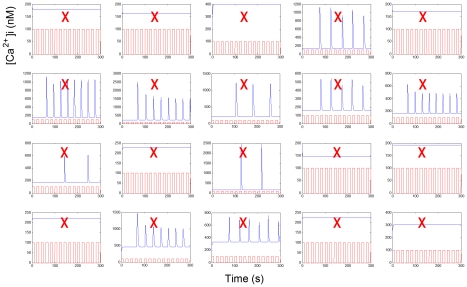
Parameter sampling cannot account for the discrepancy between mathematical models and experiments. Representative individual ‘intracellular calcium concentration vs. time’ graphs with unique parameter sets generated by the Latin Hypercube Sampling (LHS) algorithm for the Chay et al. model. Model parameters were sampled from a uniform distribution, where the minimum distribution value was one tenth of the original parameter value and the maximum distribution value was ten times the original parameter value (details provided in Materials and Methods section). Of the 500 total unique parameter sets generated by LHS, none could produce sub-threshold spikes upon periodic stimulation (red square waves), contrary to what was observed experimentally (Fig. 1, and See Fig. 1, 2 in Text S1); a red ‘X’ signifies that the parameter set did not produce sub-threshold calcium spikes.

We first analyzed the Chay et al. model [10] (Fig. 2a). As depicted in Fig. 3d, we found that as the rest period (R) between stimulation events was increased, the phase-locking ratio increased. Despite the agreement of the model with the effects of R on phase-locking ratio observed in our system (compare Fig. 1d with Fig. 3d), it could not account for the presence of sub-threshold calcium spikes (compare Fig. 1b with Fig. 3g), suggesting inaccuracies in its activation properties. We attributed the lack of sub-threshold spikes to the model mechanisms, and not model parameter values, as we used a sampling algorithm (Latin Hypercube Sampling (LHS)) to survey a range of parameter values and found no parameter set able to result in sub-threshold calcium spikes (Fig. 4). The Chay et al. model assumes that G-protein activation of PLC is a switch-like response with a Hill Coefficient of 4. Therefore if activated G-protein levels are not sufficiently high to surpass the threshold for PLC activation, a calcium spike will not result. However, the presence of sub-threshold calcium spikes in our experiments suggested that such a sharp activation threshold does not exist. While some experiments suggest that Gq-protein activation of PLC is graded [26], to our knowledge, there are no studies that have conclusively determined the nature of this interaction; furthermore, these activation properties may be cell type or signaling pathway dependent. When the Hill coefficient of the G-protein/PLC interaction was reduced below 3.5 in the Chay et al. model, calcium oscillations could not be obtained under continuous stimulation (See Fig. 4a in Text S1); furthermore, periodic stimulation of the model with Hill coefficients between 3.5 and 4 did not yield sub-threshold calcium spikes for a wide range of stimulation conditions (See Fig. 4b in Text S1). These results have important implications in terms of how extracellular chemical signals are filtered and interpreted by downstream elements. In particular, intracellular calcium is not only frequency encoded [27], but also amplitude encoded [28], which means that sub-threshold calcium responses might affect cellular responses compared to the non-responses that were noted in the Chay et al. model. Therefore, from a mechanistic standpoint, the ability to capture behaviors such as sub-threshold spikes may prove critical. In addition, these findings show that the reaction mechanisms and model parameters need to be re-evaluated for the Chay et al. model, which has been used for analysis in many other studies [5], [29], [30], [31].

Our experimental observations were then used to evaluate the Politi et al. model (Fig. 2b). Individual calcium graphs portrayed sub-threshold calcium spikes upon exposure to square-wave stimulation pulses (Fig. 3h). However, the model incorrectly predicted that larger R resulted in smaller phase-locking ratios (Fig. 3e), suggesting that the recovery properties of the model are not accurate. LHS analysis indicated that the choice of model parameter values alone could not explain these inaccuracies, suggesting that reaction mechanisms used to formulate the model needed revision.

Thus, neither of the calcium models tested was able to account for all of our experimental observations. We noted that the Politi et al. model showed continued IP3 decay between stimulation pulses, while in the Chay et al. model, IP3 levels exhibited recovery between stimulation pulses (See Fig. 5 in Text S1). In the latter model, IP3 recovery between stimulation pulses is due to a mechanism for basal IP3 production. Addition of basal IP3 production to the Politi et al. model was able to correct its deficiencies in recovery dynamics (Fig. 3 right column); the IP3 production value used in our study was similar to that of reference [32]. This model revision may provide crucial insight into physiological systems where cells or tissues require fidelity of its calcium signals to periodic chemical stimulation in order to carry out their function [33]. Accurate capture of the recovery properties of oscillatory pathways may also play a pivotal role in the entrainment of such systems [34]. We note that other mechanisms may be found that can account for our experimental observations, but basal IP3 production provides the simplest explanation and is supported by the literature [35], [36], [37]. Collectively, this would suggest that the activation and recovery mechanisms reflected in our revised Politi et al. model (positive feedback mechanism of calcium upon PLC activity, graded PLC activation by G-proteins, and basal IP3 production) are a good fit for the pathway studied here.

We also analyzed seven additional calcium oscillation models. We first explicitly included ligand-receptor-G protein dynamics in both the Chay et al. and Politi et al. models analyzed above, to test whether this would affect our predictions. Those modifications did not change the outcomes of the phase-locking analysis (phase-locking ratio vs. C, D, and R and presence of sub-threshold spikes) (See Fig. 6 in Text S1), suggesting that the discrepancy between model and experiment did not lie in the simplified way stimulation was represented in the original models. We also tested a precursor to the Chay et al. model, a model by Cuthbertson and Chay [38]. Like the Politi et al. model described above, it did not contain a basal level of protein activity, and it too yielded a descending staircase as rest period (R) was increased (See Fig. 7 in Text S1). We next tested the model developed by Atri et al. [16], and found that it produced the correct recovery behavior as well as sub-threshold spikes (See Fig. 8 in Text S1); these results can be attributed to a basal flux term and graded activation, respectively. However, the calcium oscillation dynamics of the Atri et al. model are significantly faster than the range of oscillation periods we observed experimentally. As a result, we then analyzed a version of the Li and Rinzel model [18] that features slower dynamics, as presented in the study by Sneyd et al. [5]. While the model did exhibit calcium oscillation periods closer to what we saw experimentally, it exhibited a decrease in phase-locking ratio as both C and D were increased (See Fig. 9 in Text S1). This behavior was perhaps due to an augmented inhibitory effect of calcium upon the activation of the IP3 receptor; in addition, the model did exhibit sub-threshold spikes and showed the correct recovery properties, which could be attributed to a basal flux term and graded activation, respectively. Finally, we performed phase-locking analysis on the oscillatory calcium models developed by Dupont et al. [17] and Kummer et al. [39]. The former model features feedback of calcium upon PLC activity and IP3 metabolism, similar to the Politi et al. model, and the latter model features G-protein and PLC dyanmics. While the Dupont et al. model did exhibit sub-threshold spikes, phase-locking analysis revealed that it exhibited a decrease in phase-locking ratio for increases in R (See Fig. 10 in Text S1); the Kummer et al. model exhibited sub-threshold spikes as well, but also did not show a change in phase-locking ratio with changes in C (See Fig. 11 in Text S1). Thus, although we have not performed an exhaustive search, the modified Politi et al. model developed here best describes the qualitative features of our data on the M3 pathway.

In sum, we employed a combination of microfluidics, real-time imaging, and mathematical modeling in order to probe the circuit architecture of an oscillatory signaling pathway in mammalian cells. Here chemical-induced phase-locking was explored and analysis of its properties was used to test mathematical models and elucidate molecular mechanisms. Previous reports have claimed that phase-locking events are mostly robust to mechanism details [24], [40]; this study reports that the properties of phase-locking, however, largely depend upon some of the recovery and activation properties of the molecular mechanisms of an oscillatory signaling system.

As microfluidic setups become more elaborate in their ability to generate temporal stimulation patterns, we can expect even more discriminating markers for signaling studies [41]; the diverse waveform stimulation patterns generated by microfluidic setups such as the “chemical waveform synthesizer” [42] and the “chemical signal generator” [43] should prove useful to this end. While a single optical readout (calcium) was employed for this study, the experimental setup is amenable to the use of multiple real-time readouts of cellular signaling, thereby further enhancing the number of discriminating markers for elucidation of signaling pathways. Finally, although this paper focused on calcium oscillations, we believe our approach would be well-suited for studies on various biological oscillators such as ERK [44], NFκB [45], and components involved in cell cycle [24], circadian [46], and ultradian [47] rhythms. For example, we have performed phase-locking analysis of two popular circadian oscillator models [48], [49] and seen dramatic differences in phase locking behavior between the two, despite similar behaviors under conventional stimulation conditions (See Fig. 12 in Text S1). Thus, these types of phase-locking analyses provide experimentally testable hypotheses for elucidating molecular mechanisms and show that the method is applicable to a broad range of oscillatory pathways.

## Materials and Methods

### Cell Culture

HEK293 cells were cultured in Dulbecco's Modified Eagle's Medium (DMEM) (Invitrogen) supplemented with 10% Fetal Bovine Serum (FBS) (Gibco) and were maintained at 37°C with 5% CO2 in 24-well plates. 0.25% Trypsin/EDTA (Gibco) was used to detach cells from plates and transfer them to the microfluidic setup. These cells were stably transfected with the M3 muscarinic receptor (selected with 0.4 mg/mL Geneticin (Gibco)). Cells were transiently transfected with the calcium FRET probe YC3.60 [50]. Transfections were carried out with Lipofectamine2000 (Invitrogen) using the manufacturer's protocol.

### Microfluidics

Microfluidic device molds were fabricated based upon the ones described in Futai et al. [25]. Front-side photolithography [51] was used to construct the outlet channel where cells were cultured; the remaining channels (inlets and “Braille” channels) were constructed with backside photolithography [52]. With the resulting glass mold, PDMS (1∶10 ratio of curing agent to base) was cast upon the positive relief features and allowed to cure for at least 2 hours in a 60°C oven. The resulting device was then irreversibly sealed against a thin (∼100 µm) PDMS sheet through 30 s plasma oxidation. Once sealed, the device was filled with Phosphate Buffered Saline (PBS) and sterilized for 2 hrs in a UV oven. To ensure cell adhesion, the chip was subsequently filled with 100 µg/mL laminin (Invitrogen) and allowed to incubate at 37°C for two hours. After this, the chip was flushed and refilled with DMEM supplemented with 10% FBS. Transfected HEK293 cells were then seeded from the outlet port and were appropriately positioned in the outlet hydrodynamically. The cells were then allowed to attach overnight.

A custom program written in Visual Basic was used to control the dynamic pumping mediated by Braille-actuation [53], and thereby create the various temporal stimulation patterns used in experiments (Fig. 1a); experiments with fluorescein solution confirmed the nearly square-wave shape and reproducibility of these patterns. Carbachol (CCh) dissolved in imaging media [54] was added to one of the inlet reservoirs, and the other reservoir was filled with stimulant-free imaging media. Cells in the devices were maintained at 37°C via a transparent indium tin oxide heater [55], situated between the objective and the thin PDMS-sheet upon which the cells were cultured. Fluid flow did not elicit detectable intracellular calcium responses.

### Imaging

Cells were imaged with a TE2000-U Nikon inverted microscope, using a 20× objective, a standard 100W mercury lamp, and a 490 nm long pass dichroic mirror. A CoolSnap HQ2 camera (Photometrics, Tucson, AZ) was used to capture fluorescence images of YC3.60-transfected cells. Cells were excited at 450 nm and the emission signals were captured at 490 and 535 nm (filters from Chroma Technology Corp, Rockingham, VT). An ND4 neutral density filter was used to reduce photo-bleaching. The excitation and emission filter wheels were controlled by the Lambda 10-3 Shutter Controller (Sutter Instruments, Novato, CA). Images were acquired every 3 s, and an exposure time of 100 ms was used. The program MetaFluor (Molecular Devices, Downington, PA) was used for image acquisition and processing; for each emission image (at 490 nm and 535 nm) the background was subtracted, ratiometric images were constructed (intensity at 535 nm/intensity at 490 nm), and calcium FRET ratios of individual cells were generated with this software. These FRET ratios (I) were normalized by the minimum FRET ratio obtained in the experimental run (I_0_), and accordingly I/I_0_ was plotted in our figures, as has been done previously [56].The normalized ratio values of the calcium peaks fell between 1.2 and 7.5, which was in accord with previously obtained values using the same FRET indicator [50].

The resulting images were then analyzed to calculate the phase-locking ratios by dividing the number of calcium spike events by the number of CCh stimulation inputs. Since at least several cells always responded to a particular stimulation pulse, we concluded that when cells did not respond, it was due to phase-locking and not a malfunction with the microfluidic setup (Video S1).

### Computation of Phase-Locking Ratios

Cells were exposed to 9–18 stimulation inputs, and the number of calcium responses for each run was recorded. For instance, for a cell that had been exposed to 12 CCh stimulation pulses and responded with 6 calcium spikes, the phase-locking ratio was computed as 0.5. Calcium spikes that were above levels of background noise (typically more than 10% maximum calcium spike height) but did not reach an amplitude greater than 33% of the maximum calcium spike height were not counted as true calcium spikes and were deemed sub-threshold calcium spikes (See Fig. 1 and Fig. 2 in Text S1). Phase-locking ratios were computed for individual cells, and averages and standard errors of the mean were computed for each experimental condition. Statistics were based upon three experiments (each of no less than 20 cells) for each experimental condition. Between 85–106 cells were examined for each experimental condition. The unpaired Student t-test was used to statistically compare pairs of experimental conditions; p<0.05 was used as a threshold of statistical significance.

### Mathematical Models

Nine mathematical models of oscillatory calcium signaling were evaluated in our study: the Chay et al. model [10] (Fig. 2a), the positive feedback Politi et al. model [11] (Fig. 2b), the Cuthbertson and Chay model [38], the Li and Rinzel model [13], the Atri et al. model [16], the Chay et al. and Politi et al. models with ligand/receptor/G-protein dynamics from Ref. [57], the Dupont et al. model [17], and the Kummer et al. model [39]. For all these mathematical models, we used the equations and initial conditions defined in the original publications (except for the Li and Rinzel model, for which we used the adaption developed in Sneyd et al. [5]); model equations, parameters, initial conditions, and brief model descriptions for all models used in this study are provided in Text S1, starting on page 13. For the Chay et al. model, it was assumed that receptor-mediated G-protein activation was proportional to stimulant concentration. For the Politi et al. model, it was assumed that the maximal rate of PLC-mediated IP3 production was proportional to stimulant concentration. These assumptions are based upon those from the original publications. For the Politi et al. model, we used calcium flux strength ε = 5 to reflect the role of extracellular flux in calcium oscillations [58]. The mathematical systems were exposed to 12 square-wave stimulation pulses and the corresponding number of calcium spike responses was counted in order to compute phase-locking ratios; the criteria for assessing the phase-locking ratio were the same as those for experiments, as described earlier in the Materials and Methods Section. To assess the effect of rest period on the phase-locking ratio, this parameter was varied, while stimulant concentration and stimulation duration were fixed; we then plotted the resulting phase-locking ratio against the rest period (Fig. 3- middle row). The same procedure was applied to assess the effects of stimulant concentration and stimulation duration on the phase-locking ratio, respectively (See Fig. 3 in Text S1). Stimulation parameters for the mathematical models were chosen such that the range in behaviors under periodic stimulation matched those observed in experiments. The stimulation concentration ‘C’ is represented differently for each model, as noted in Text S1.

Original parameters were used for both circadian models [48], [49].

All models were coded in MATLAB version 7.8.0 (MathWorks Inc, Natick, MA) and the system of ODEs was solved with the stiff solver ode15s.

### Latin Hypercube Sampling

We used Latin Hypercube Sampling (LHS) to check if inaccuracies in model parameter values alone could account for differences between experimental results and model predictions. LHS is a highly effective method for exploring parameter spaces for mathematical models [59], [60], [61], [62]. Using LHS code from Marino et al. [60] (http://malthus.micro.med.umich.edu/lab/usadata/), we varied model parameter values by sampling from a normal distribution with a 25% standard deviation; original parameter values were used as the mean. Larger standard deviations (100%) did not yield results different from those at 25% standard deviation. We also sampled parameters from a uniform distribution; the boundaries of the distribution were set by using one tenth of the original parameter value as the minimum and ten times the original parameter value as the maximum. As was the case for sampling from a normal distribution, sampling from a uniform distribution did not yield any parameter sets that could account for the discrepancies between models and experiments. For the Chay et al. model, we varied all twelve independent parameters; for the Politi et al. model, we varied all 17 independent parameters, except for β, which represented the ratio of ER to cytoplasm volume. LHS was run for 500 iterations on each model, and each model output was analyzed to decipher whether the results matched experimental observations (either by constructing ‘phase-locking ratio vs. rest period’ graphs for the Politi et al. model or by looking at individual model runs for the Chay et al. model, as depicted in Fig. 4).

## Supporting Information

Text S1 Figures 1–12, mathematical model equations, parameters, initial conditions, and brief descriptions for all models used in the study.
(0.93 MB PDF)

Video S1 Movie of HEK293 cell with phase-locking ratio of 0.5 (cell ‘A’), and neighboring cells with 1∶1 calcium phase-locking (‘B’).
(1.37 MB MPG)
