# Supplementary material for: Phase-Locked Signals Elucidate Circuit Architecture of an Oscillatory Pathway
Source: PLoS Comput Biol. 2010 Dec 23;6(12):e1001040. doi: 10.1371/journal.pcbi.1001040 (PMC3009597; doi:10.1371/journal.pcbi.1001040)
Supplement: Text S1 — Figures 1–12, mathematical model equations, parameters, initial conditions, and brief descriptions for all models used in the study. (0.93 MB PDF) [file pcbi.1001040.s001.pdf]

Text S1: Figures, Mathematical Modeling

Supplemental Figures

Fig. 1: Representative cases of individual intracellular calcium signals vs. time graphs for cells addressed with periodic carbachol stimulation (C = 25 nM, D = 24 s, R = 24 s). I/I_0 is the normalized FRET ratio of the intracellular calcium signals, as was used in Fig. 1. The '(-)' symbols denote skipped beats, based upon the criteria explained in detail in the Materials and Methods Section. Sub-threshold spikes are apparent in a majority of the graphs.

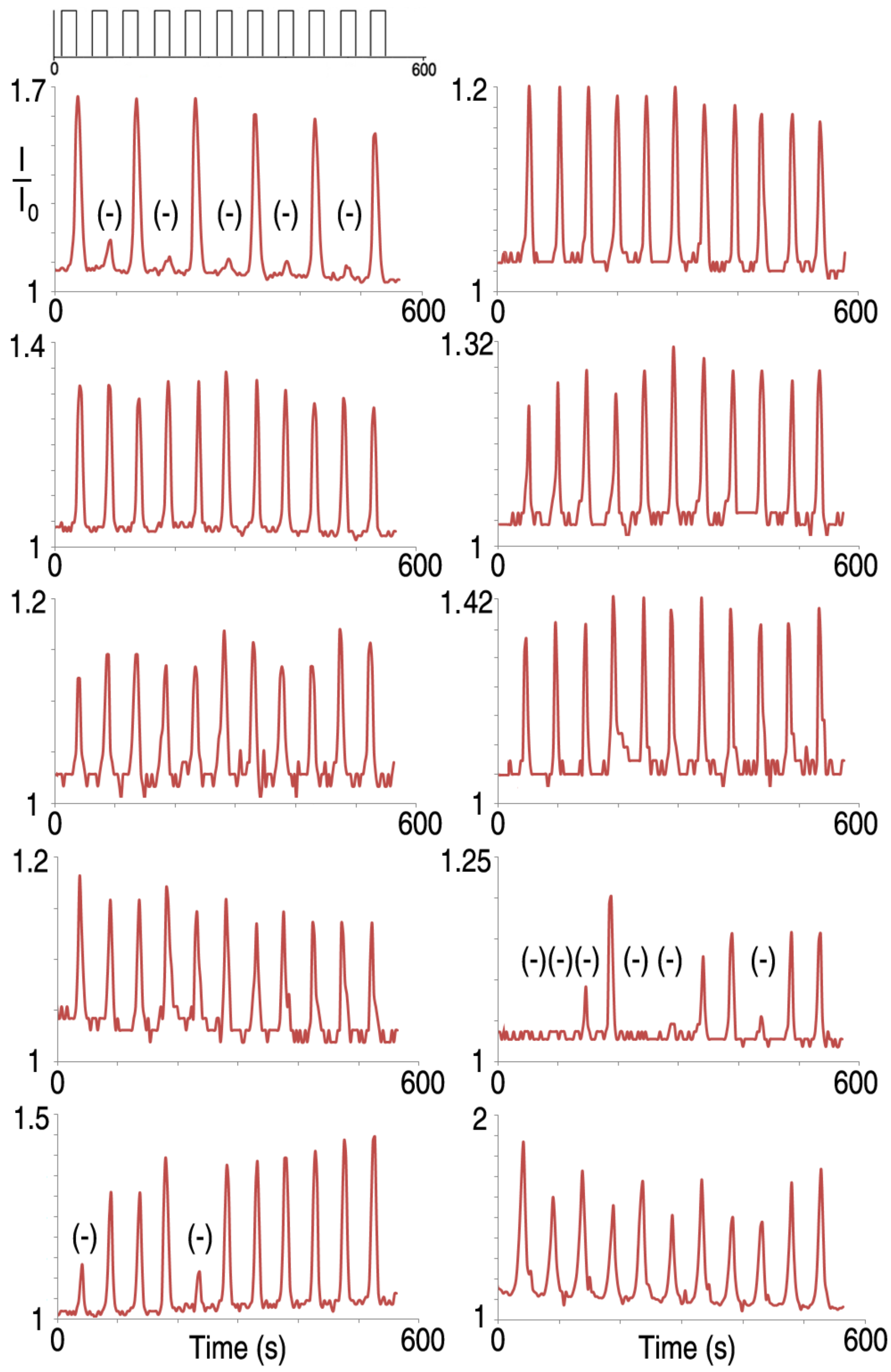

Fig. 2: Representative cases of individual intracellular calcium signals vs. time graphs for cells addressed with periodic carbachol stimulation (C = 10 nM, D = 24 s, R = 24 s). I/I_0 is the normalized FRET ratio of the intracellular calcium signals, as was used in Fig. 1. The '(-)' symbols denote skipped beats, based upon the criteria explained in detail in the Materials and Methods Section. Sub-threshold spikes are apparent in a majority of the graphs. A greater proportion of cells exhibit skipped beats compared to Text S1 Fig. 1, since the stimulant concentration is lower.

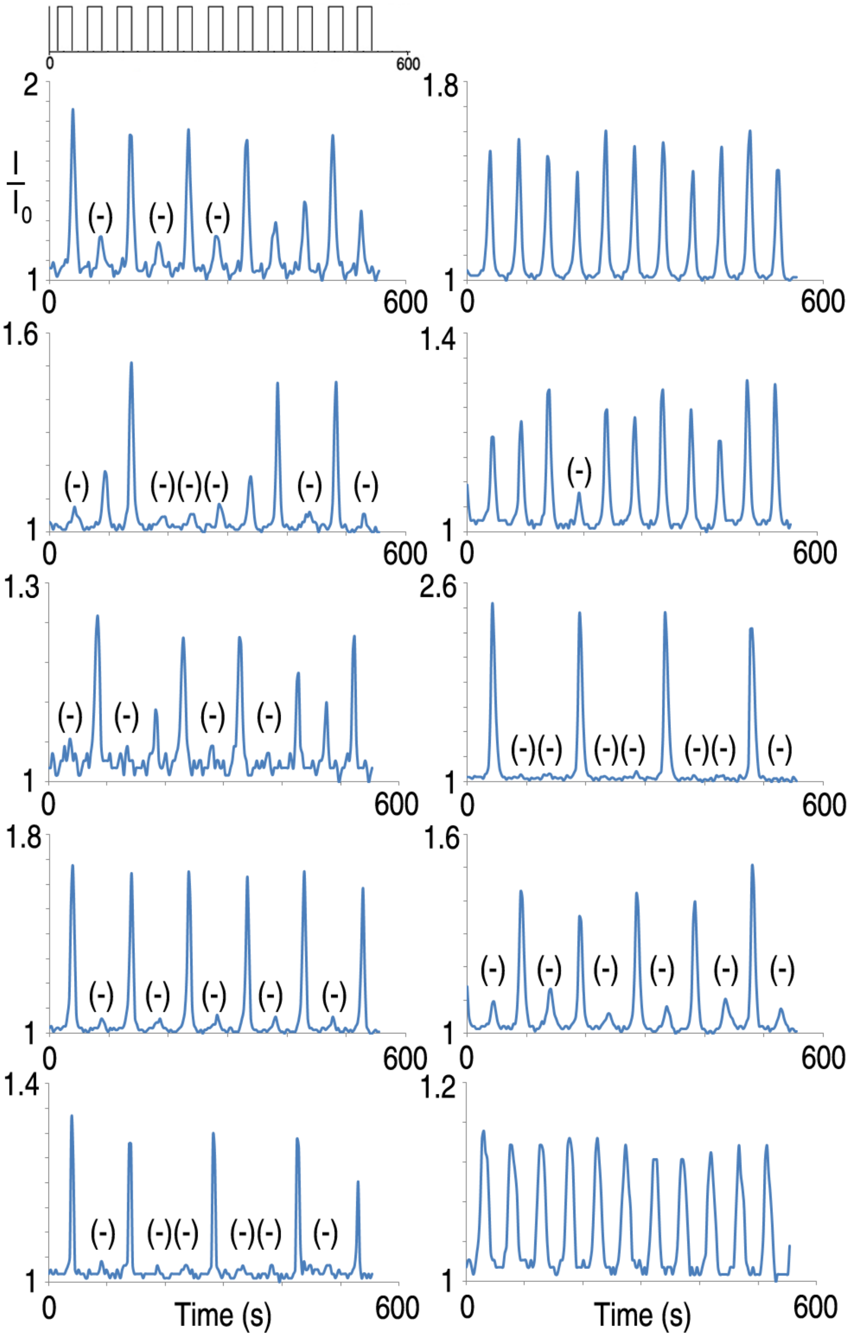

Fig. 3: Phase-locking behaviors: experimental results (a, b) and theoretical results (c-h) for the Chay et al. model (c, f), the positive feedback Politi et. al. (d, f) (middle column) and the revised Politi et al. model (basal PLC activity = 0.3 μM/s) (e, g) when stimulant concentration (C) and stimulation duration (D) were varied. a) Phase-locking ratio vs. C, with D and R = 24 s. b) Phase-locking ratio vs. D, with C = 10 nM and R = 24 s. c)-e) Phase-locking Ratio vs. Stimulant Concentration. In c) the stimulant concentration has units of 1/s and represents the rate of receptor-mediated G-protein activation. For d) and e) the stimulant concentrations have units of μM/s and represent the maximal rate of IP3 production. For c)-e), D = 10 s and R = 50 s. f)-h) Phase-locking ratio vs. D with R fixed at 60 s. For f) C = 0.03 1/s, for g) C = 0.8 μM/s, and for h) C = 0.3 μM/s. Bars indicate the S.E.M., representative of three experiments for each experimental condition; for each experiment, the responses of least 20 cells were recorded. All pairs of experimental conditions were statistically significant as determined by the Student t-test ($p<0.05$).

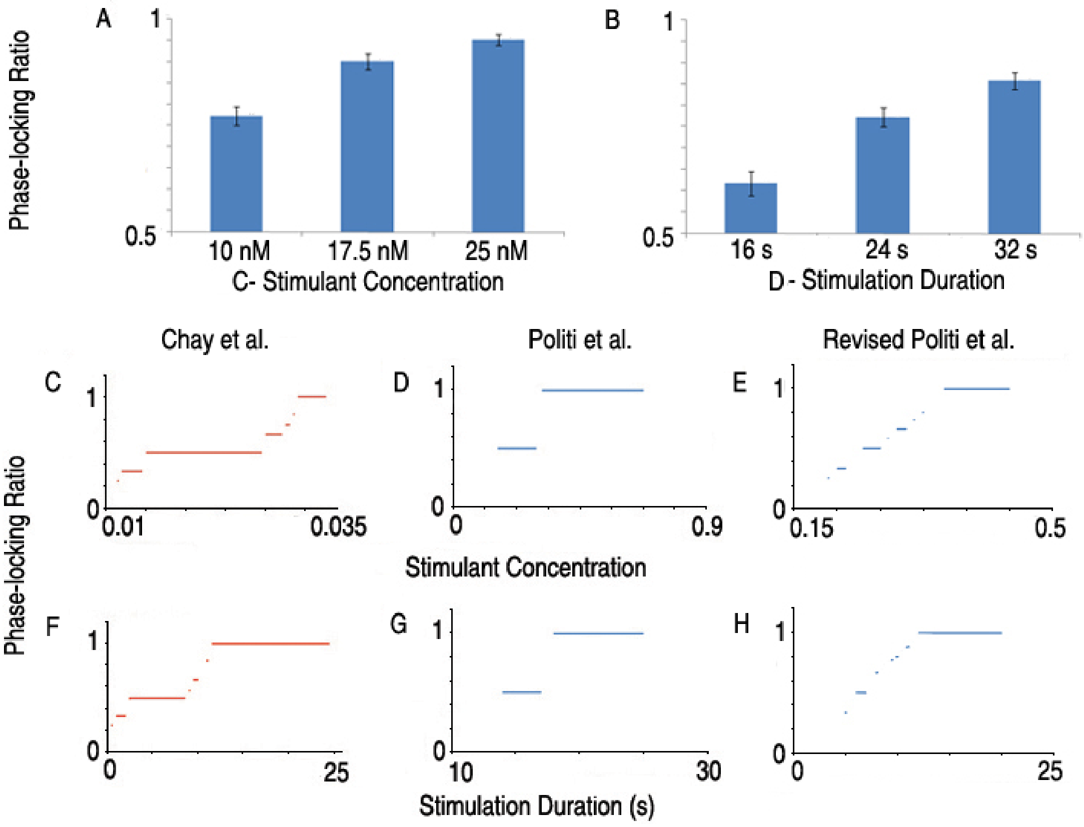

Fig. 4: a) Continuous stimulation of the Chay et al. model with Hill coefficients less than 3.5 for Gq-mediated PLC activation. The system does not exhibit any calcium oscillations under this condition. b) For Hill coefficients between 3.5 and 4, sub-threshold spikes are not observed upon periodic stimulation. Depicted is a representative trace of calcium concentration vs. time under periodic stimulation conditions (C = 0.02 1/s , D = 50 s, R = 50 s, Hill Coefficient = 3.5). For all conditions tested, the original model parameters were used.

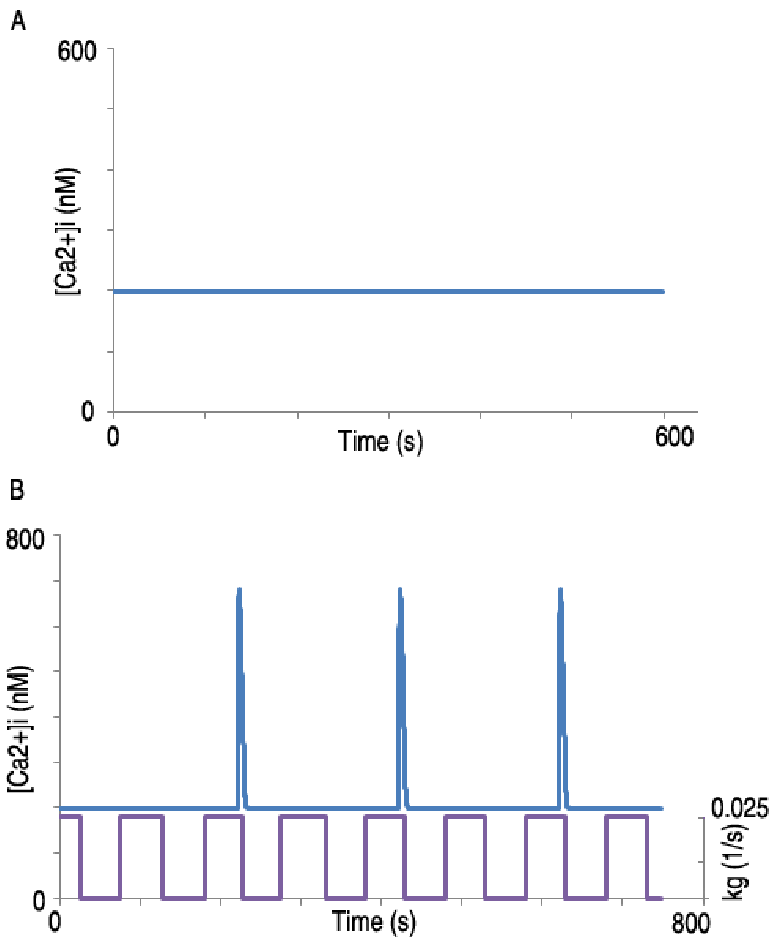

Fig. 5: IP3 recovery dynamics for the positive feedback Politi et al. model (purple), for the Chay et al. model (red), and the former model with basal PLC activity (blue). The times and magnitudes of the IP3 curves have been offset for easier comparison. For each curve, the stimulation duration was 10 s and the rest period was 500 s. The stimulant concentration was 0.05 1/s for the Chay et al. model, 1.2 μM/s for the Politi et al. model, and 0.3 μM/s for the latter model with basal IP3 production. Basal IP3 production was set at 0.3 μM/s.

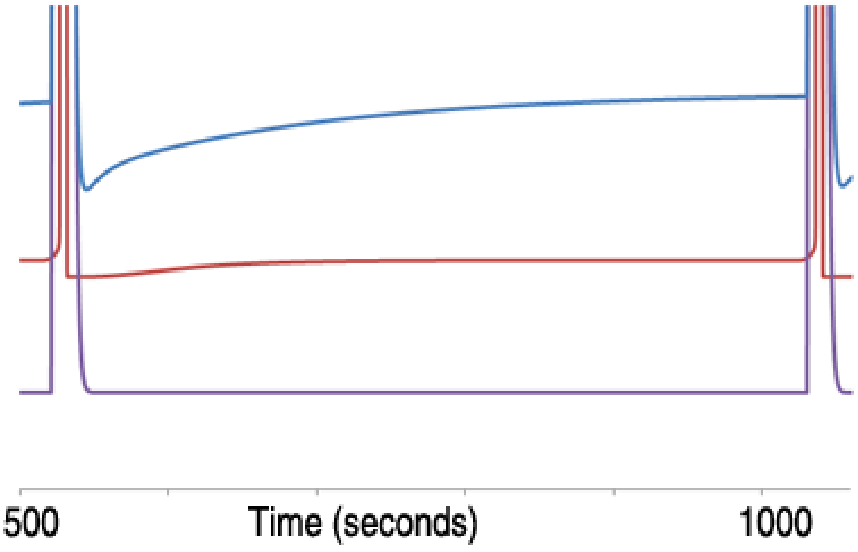

Fig. 6: Phase-locking analysis of the Chay et al. (left) and Politi et al. (right) models supplemented with ligand, receptor, and G-protein dynamics. a) Calcium oscillation period vs. C (stimulant concentration). b) Phase-locking ratio vs. C, with D = 25 s and R = 25 s. c) Phase-locking ratio vs. D, with C = 55 nM (left), 15 nM (right) and R = 25 s. d) Phase-locking ratio vs. R, with C = 55 nM (left), 15 nM (right) and D = 25 s. e) Individual calcium signal vs. time graphs, with C = 55 nM (left), 15 nM (right), D = 25 s, and R = 25 s. Parameter values and rate equations for the ligand, receptor, G-protein dynamics were taken from [1,2]. Notably, the behaviors of the Chay et al. and Politi et al. models with enhanced biochemical detail under periodic stimulation are similar to the original models (Fig. 3 and Text S1 Fig. 3).

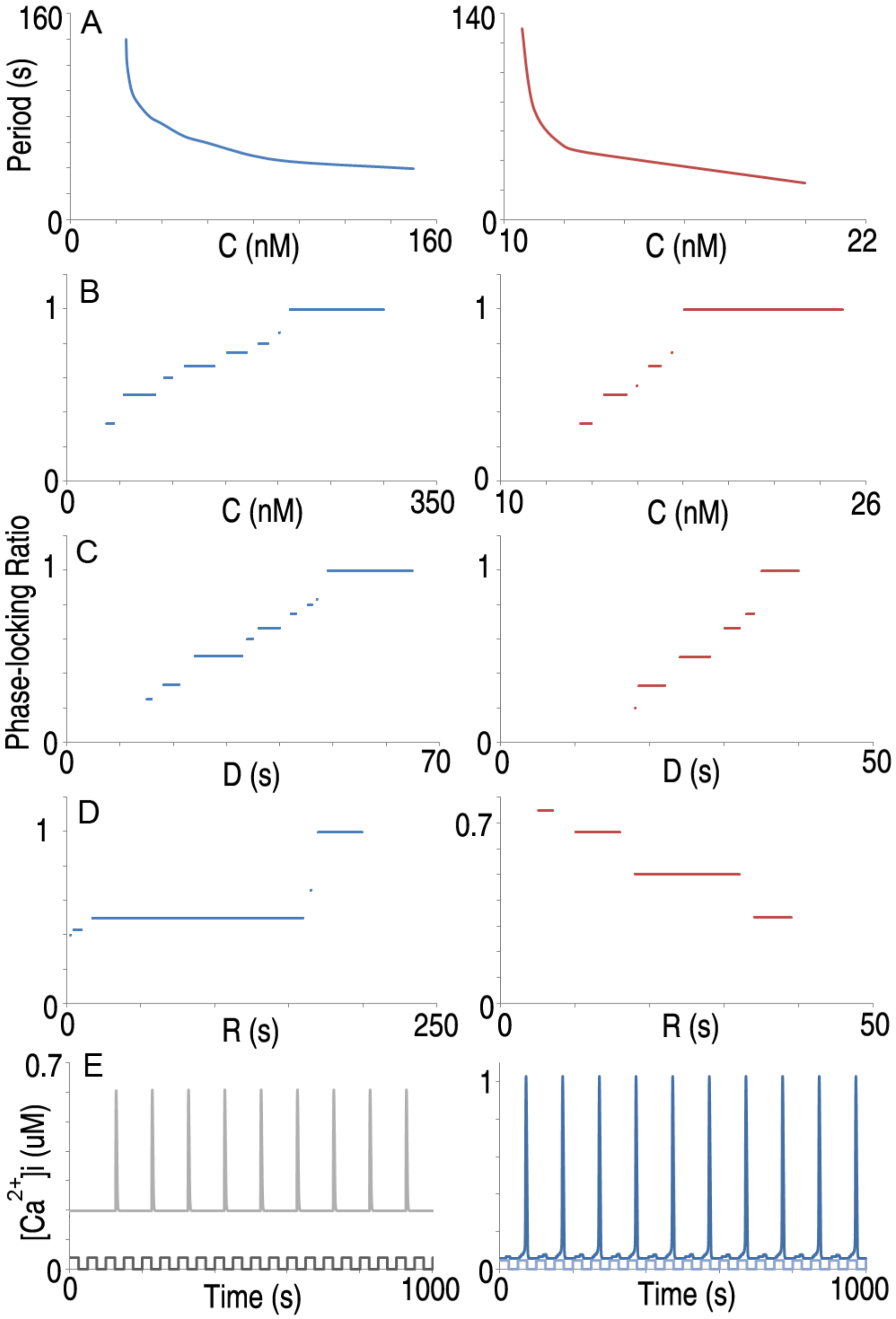

Fig. 7: Phase-locking analysis of the Cuthbertson and Chay model. a) Calcium oscillation period vs. C (stimulant concentration). b) Phase-locking ratio vs. C, with D = 25 s and R = 25 s. c) Phase-locking ratio vs. D, with C = 0.015 1/s and R = 25 s. d) Phase-locking ratio vs. R, with C = 0.015 1/s and D = 25 s. e) Individual calcium signal vs. time graph with the following periodic stimulation parameters: C = 0.015 1/s, D = 25 s, and R = 25 s. In d), the phase-locking ratio decreases for increases in rest period, and in e), there is an absence of sub-threshold spikes.

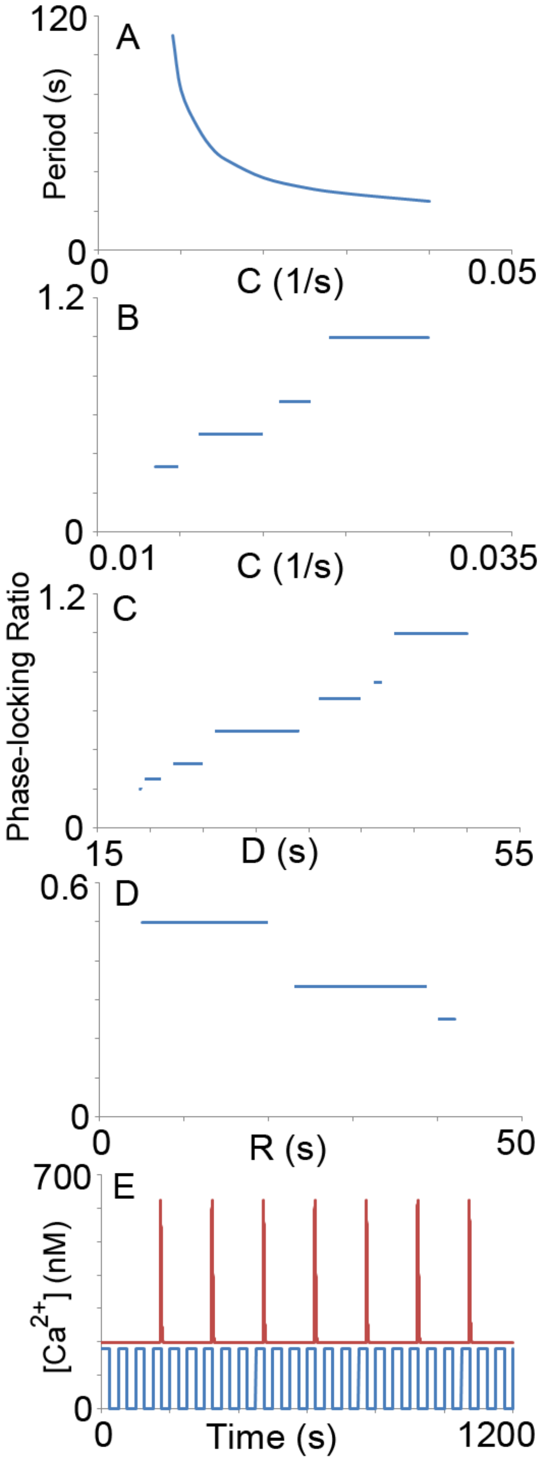

Fig. 8: Phase-locking analysis of the Atri et al. model. a) Calcium oscillation period vs. C (stimulant concentration). b) Phase-locking ratio vs. C, with D = 10 s and R = 3 s. c) Phase-locking ratio vs. D, with C = 0.57 and R = 3 s. d) Phase-locking ratio vs. R, with C = 0.57 and D = 10 s. e) Individual calcium signals vs. time graph, with the following periodic stimulation parameters: C = 0.57, D = 10 s, and R = 3 s. The model predicts all the correct behaviors seen experimentally under periodic stimulation, with the caveat that the calcium oscillation dynamics are much faster than what was observed experimentally.

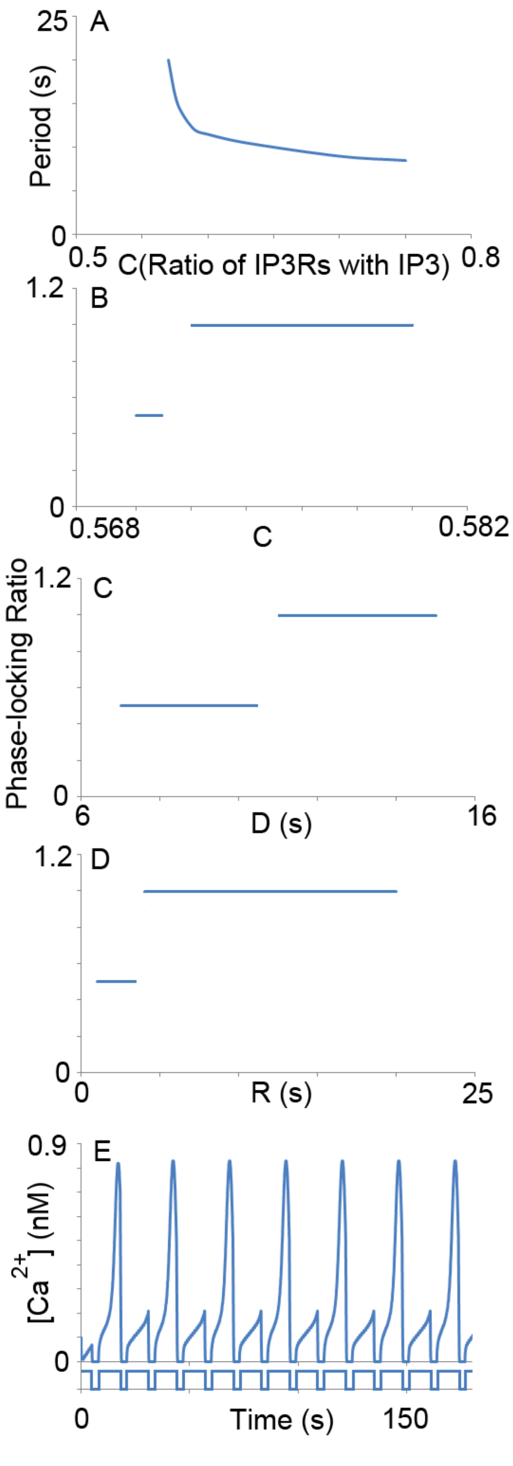

Fig. 9: Phase-locking analysis of the Li and Rinzel Model (a version adapted for the study by Sneyd et al. [3]). a) Calcium oscillation period vs. C (stimulant concentration). b) Phase-locking ratio vs. C, with D = 20 s and R = 20 s. c) Phase-locking ratio vs. D, with C = 0.4 µM/s and R = 20 s. d) Phase-locking ratio vs. R, with C = 0.4 µM/s and D = 20 s. e) Individual calcium signal vs. time graph, with the following periodic stimulation parameters: C = 0.4, µM/s D = 20 s, R = 20 s. In b) and c), the phase-locking ratio decreases with increases in C and D, opposite of what was observed experimentally.

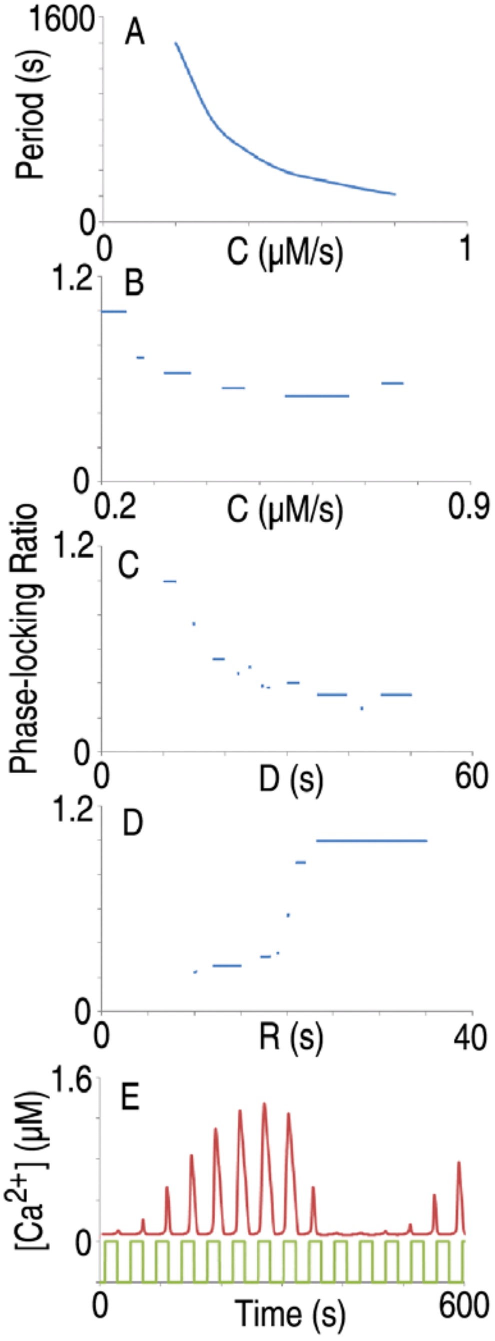

Fig. 10: Phase-locking analysis of the Dupont et al. model. a) Calcium oscillation period vs. C (stimulant concentration). b) Phase-locking ratio vs. C, with D = 1 s and R = 2 s. c) Phase-locking ratio vs. D, with C = 0.28 and R = 2 s. d) Phase-locking ratio vs. R, with C = 0.28 and D = 1 s. e) Individual calcium signal vs. time graph, with the following stimulation parameters: C = 0.28 1/s, D = 1 s, and R = 2 s. In d), the phase-locking ratio decreases with increases in R, which was not observed experimentally.

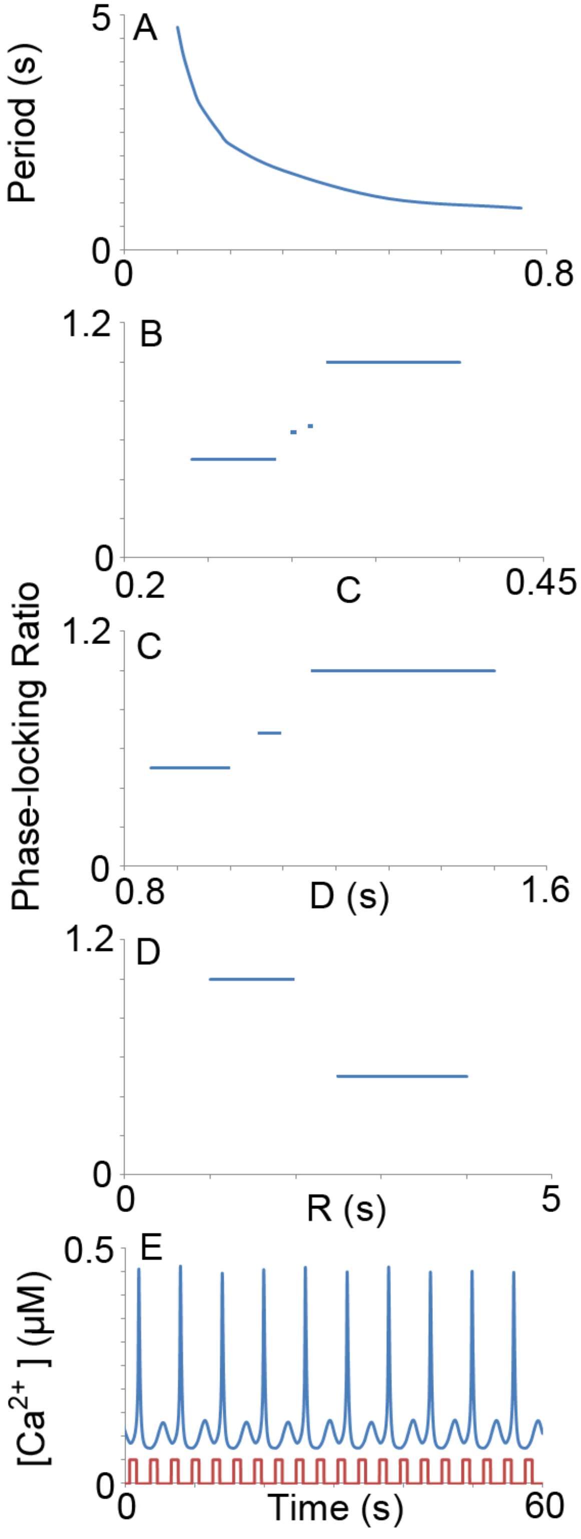

Fig. 11: Phase-locking analysis of the Kummer et al. model. a) Calcium oscillation period vs. C (stimulant concentration. b) Phase-locking ratio vs. C, with D = 4 units and R = 1 unit. c) Phase-locking ratio vs. D, with C = 1.3 units and R = 1 unit. d) Phase-locking ratio vs. R, with C = 1.3 units and D = 4 units. e) Individual calcium signal vs. time graph, with the following stimulation parameters: C = 1.3 units, D = 5.5 units, R = 1 unit. In b), the phase-locking ratio remains constant with increases in C, which was not observed experimentally; in d), the phase-locking ratio decreases to zero and then increases with increases in R, which was also not observed experimentally.

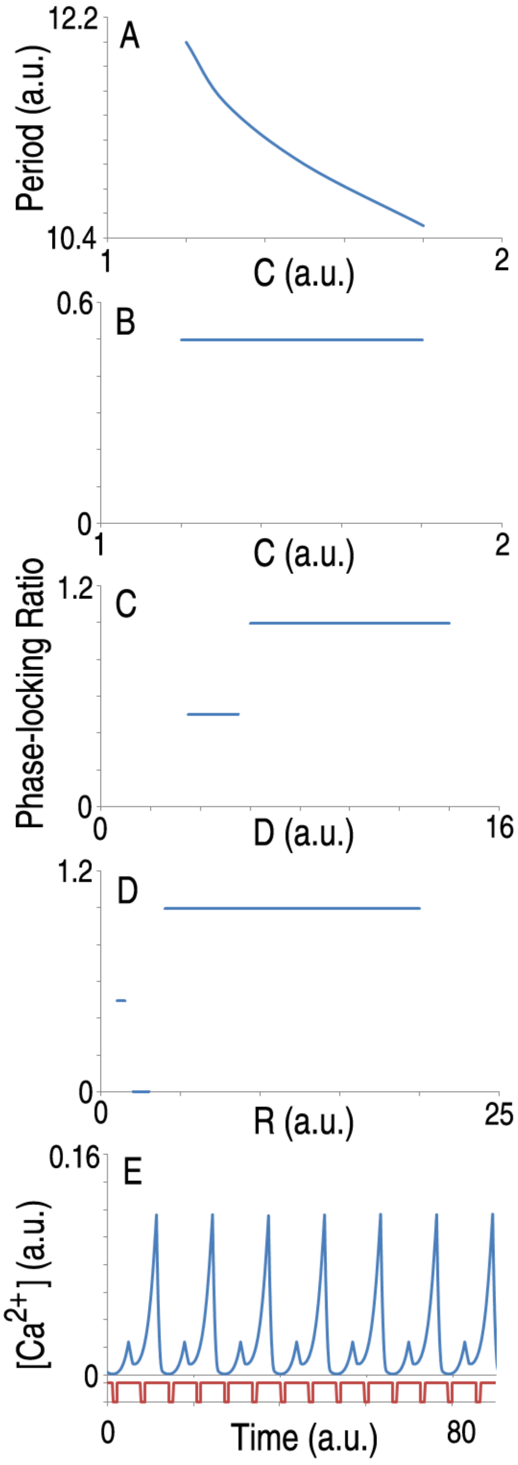

Fig. 12: Phase-locking analysis of two models of Circadian rhythms: the Tyson et al. model [4] and the Goldbeter model [5]. a) Plotting the phase-locking ratio vs. R for the Tyson et al. model revealed that for small stimulation durations (D), the phase-locking ratio increased, then decreased to zero. The stimulation parameters used to generate this graph were: C = 1 Cm/hr and D = 2 hrs. For larger D, it was found that the phase-locking ratio increased, and remained at the value one, suggesting that the recovery properties of the Tyson et al. model depend partly on D. b) For the Goldbeter model, an increase in R resulted in a corresponding increase in the phase-locking ratio for both small and large D. These results suggest that the recovery properties of the Goldbeter model do not depend on D. The following stimulation parameters were used to generate the graph depicted: C = 2 μM/hr and D = 2 hrs.

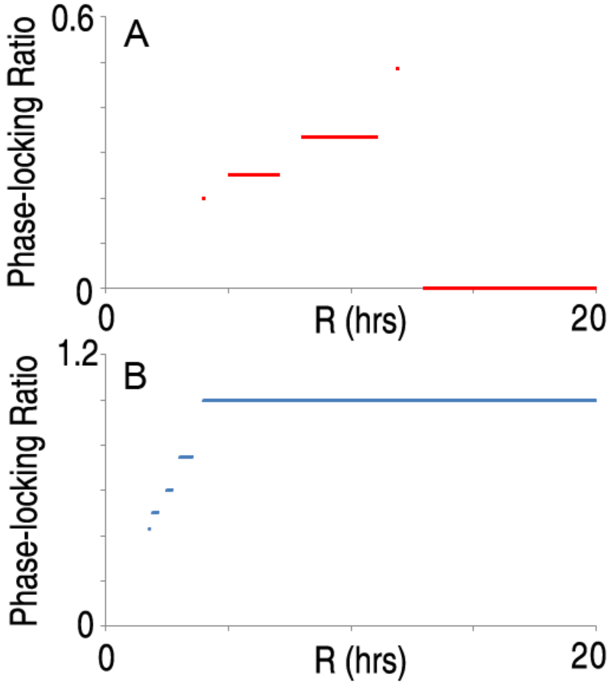

Mathematical Modeling

The following section contains all the model equations, parameters, and initial conditions for the 9 mathematical models of oscillatory calcium signaling analyzed in this study; also included are brief descriptions of each model. Additional model details can be found in the original publications; references are provided to direct the reader to these works. The model equations, parameters, and initial conditions for the two circadian models analyzed in this study are also provided in this section.

A. Chay et al. model (Reference [6])

i. Model Description:

The Chay et al. model is based on experimental observations that calcium oscillation periods vary from seconds to minutes and the amplitude of the calcium signals does not change drastically with changes in stimulant concentration. Calcium oscillations result from a feedback loop involving build-up of activated G-proteins, switch-like PLC activation, IP3/diacylglycerol release, calcium release, followed by negative feedback by DAG upon G-protein activation. It was the first model to be used to theoretically analyze the effect of periodic stimulation upon calcium signaling.

ii. Model equations:

(1)

$$\frac{d[G \propto GTP]}{dt} = (kg + basal) * (G0 - [G \propto GTP] - 4 * [PLC]) - 4 * kp * [G \propto GTP]^4 * (P0 - PLC) - hg * [G \propto GTP]$$

(2)

$$\frac{d[IP3]}{dt} = kd * [PLC] - hd * [IP3] + ld$$

(3)

$$\frac{d[Ca^{2+}]}{dt} = \rho kc * (\frac{[IP3]^3}{Ks^3 + [IP3]^3}) - \rho * [Ca^{2+}] + \rho lc$$

(4)

$$\frac{d[PLC]}{dt} = kp * [G \propto GTP]^4 * (P0 - PLC) - hp * [PLC]$$

(5)

$$kn = kn' * \left(\frac{[DAG]^2}{Kd^2 + [DAG]^2}\right); \quad \text{where } kn = kp, hp, kd$$

Rate equations for the ligand/receptor dynamics used for Text S1 Fig. 6:

(6)

$$\frac{d[R]}{dt} = kr * [LR] - hr * (R0 - [LR]) * L$$

(7)

$$\frac{d[LR]}{dt} = hr * (R0 - [LR]) * L - kr * [LR]$$

The equation describing activated G-protein dynamics was changed to the following:

(8)

$$\frac{d[G \propto GTP]}{dt} = kg2 * ([LR] + basal2) * (G0 - [G \propto GTP] - 4 * [PLC]) - 4 * kp * [G \propto GTP]^4 * (P0 - PLC) - hg * [G \propto GTP]$$

$[G \propto GTP]$ = activated G-protein; $[IP3]$ = inositol triphosphate; $[Ca^{2+}]$ = intracellular calcium; $[PLC]$ = phospholipase C; $[R]$ = receptor; $[LR]$ = ligand-receptor complex; [DAG] = diacylglycerol = [IP3] (in the original publication, it was assumed that these two components are produced in equimolar quantities)

iii. Parameter Table:

Parameter	Description	Value
kg	G-protein activation rate constant (activated receptor contribution)	Varied (units: 1/s)
hg	G-protein hydrolysis rate constant	0 (units: 1/s)
hd	IP3 hydrolysis rate constant	100 1/s
ld	IP3 leak rate	250 nM/s
ρkc	Calcium activation rate by IP3	$9*10^4$ nM/s
ρhc	Calcium removal rate constant	1 1/s
ρlc	Calcium leak rate	200 nM/s
Ks	Half-activation constant for IP3	300 nM
Kd	Half-activation constant for DAG	25 nM
basal	Basal G-protein activation rate	0.005 1/s

G0	Total G-protein Concentration	200 nM
P0	Total PLC Concentration	10 nM
R0[1]	Total Receptor Concentration	2000 nM
kd’	DAG-mediated PLC activation rate constant	700 1/s
kp’	DAG-mediated G-protein/PLC dissociation rate constant	$2*10^{-7}$ 1/(nM^4*s)
hp’	DAG-mediated PLC deactivation rate constant	0.5 1/s
kr[2]	Ligand-receptor dissociation rate constant	0.5 1/s
hr[2]	Ligand-receptor association rate constant	0.004 1/(nM*s)
kg2[1]	G-protein activation rate from ligand-receptor contribution	$3.9*10^{-5}$ 1/(nM*s)
basal2[1]	Basal ligand-receptor concentration	130 nM

[1] values not in original model; Note: multiplication of parameters ‘kg2’ and ‘basal2’ yield a basal stimulation term with the same value and units as the parameter ‘basal’ from the original publication.

[2] values taken from [1]

iv. Initial Conditions:

$[\mathrm{G} \propto \mathrm{GTP}](0) = 0$; $[\mathrm{IP3}](0) = 0$; $[\mathrm{Ca}^{2+}](0) = 200$ nM; $[\mathrm{PLC}](0) = 0$; $[\mathrm{R}](0) = 0$; $[\mathrm{LR}](0) = 0$

For the original Chay et al. model, the parameter ‘kg’ was used to represent the stimulant concentration ‘C’ (as was done in the original publication). For the Chay et al. model that included ligand-receptor dynamics, the parameter ‘L’ represented ‘C’.

B. Politi et al. model (Reference [7])

i. Model Description:

The Politi et al. is able to produce calcium oscillation periods on the order of seconds to minutes, similar to what was observed experimentally in our studies. The model features calcium feedback upon IP3 metabolism, a key feature that was found to expand the range of oscillation periods. Oscillations in this model are produced by a feedback scheme whereby IP3 results in calcium release, and calcium then enhances its own release and IP3 production, but at high concentrations, calcium inhibits its own release. Several features of the model were experimentally validated.

ii. Model Equations:

(1)

$$\nu plc = (Vplc + basal) * \left(\frac{[Ca^{2+}]^2}{Kplc^2 + [Ca^{2+}]^2}\right)$$

(2)

$$\nu deg = (k5p + k3k * \left(\frac{[Ca^{2+}]^2}{K3k^2 + [Ca^{2+}]^2}\right)) * [IP3]$$

(3)

$$\nu rel = \left(k1 * \left([IP3r] * \frac{[Ca^{2+}]}{Ka + [Ca^{2+}]} * \frac{[IP3]}{Kp + [IP3]}\right)^3 + k2\right) * ([Ca^{2+}(ER)] - [Ca^{2+}])$$

(4)

$$\nu serca = Vserca * \left(\frac{[Ca^{2+}]^2}{Kserca^2 + [Ca^{2+}]^2}\right)$$

(5)

$$\nu in = \nu 0 + \emptyset * Vplc * (\frac{1}{k3k + k5p})$$

(6)

$$\nu out = Vpm * \left(\frac{[Ca^{2+}]^2}{Kpm^2 + [Ca^{2+}]^2}\right)$$

(7)

$$\frac{d[IP3]}{dt} = \nu plc - \nu deg$$

(8)

$$\frac{d[Ca^{2+}]}{dt} = \nu rel - \nu serca + \varepsilon * (\nu in - \nu out)$$

(9)

$$\frac{d[Ca^{2+}(ER)]}{dt} = \left(\frac{1}{\beta}\right) * (\nu serca - \nu rel)$$

(10)

$$\frac{d[IP3r]}{dt} = \left(\frac{1}{Tr}\right) * (1 - [IP3r] * \frac{Ki + [Ca^{2+}]}{Ki})$$

Ligand/Receptor/G-protein dynamics for this model (used in Text S1 Fig. 6) were taken from [2]:

(11)

$$\frac{d[LR]}{dt} = k1' * [R] * [L] - k2' * [LR]$$

(12)

$$[R] = [R(total)] - [LR]$$

(13)

$$\frac{d[G \propto GTP]}{dt} = k3 * [G] - k4 * [G \propto GTP] + k5 * [G] * [LR] - k6 * [PLC] * [G \propto GTP]$$

(14)

$$\frac{d[G \propto GDP]}{dt} = k4 * [G \propto GTP] + k4 * [PLC(active)] - k7 * [G \propto GDP][\beta\gamma]$$

(15)

$$[G] = [G(total)] - [G \propto GTP] - [G \propto GDP] - [PLC(active)]$$

(16)

$$[\beta\gamma] = [G \propto GTP] + [G \propto GDP] + [PLC(active)]$$

(17)

$$\frac{d[PLC(active)]}{dt} = k6 * [PLC] * [G \propto GTP] - k4 * [PLC(active)]$$

(18)

$$[PLC(total)] = [PLC] + [PLC(active)]$$

(19)

$$Vplc = k8 * [PLC(active)]$$

[IP3] = inositol triphosphate; $[Ca^{2+}]$ = intracellular calcium; $[Ca^{2+}(ER)]$ = endoplasmic reticulum calcium; [IP3r] = inositol triphosphate receptors not inactivated by calcium; [LR] = ligand-receptor complex; [GαGTP] = activated G-

protein; [GαGDP] = inactivated G-protein; [G] = non-activated/non-inactivated G-proteins; [βγ] = G-protein [βγ] subunits; [PLC(active)] = activated phospholipase C; vplc = rate of IP3 synthesis by PLC; vdeg = rate of IP3 degradatin by IP3 3-kinase and/or IP3 5-phosphatase; vrel = calcium flux through IP3 receptors; vout = rate of active transport of calcium across the plasma membrane; vserca = rate of active transport of calcium across the ER; vin = calcium influx due to stimulation and leaking

iii. Parameter Table for the Politi et al. model:

Parameter	Description	Value
Vplc	Activated PLC-mediated IP3 production rate constant	Varied for original model (units: µM/s)
K3k	Half-activation constant of IP3K	0.4 µM
k5p	IP3 dephosphorylation rate constant	0.66 1/s
β	Ratio of effective volumes of the ER/cytosol	0.185
Kserca	Half-activation constant for SERCA pump	0.1 µM
Kpm	Half-activation constant for PMCA pump	0.12 µM
ϕ	Stimulation-dependent influx	0.0047 1/s
Ctot	Total Calcium concentration	2 µM
k2	Calcium leak	0.0203 1/s
Ki	Calcium binding to inhibiting site	0.4 µM
Tr	Characteristic time of IP3r inactivation	12.5 s
k3k	IP3 phosphorylation rate constant	0
Kplc	Half-activation constant of PLC	0.2 µM
Vserca	Maximal SERCA pump rate	0.9 µM/s
Vpm	Maximal PMCA pump rate	0.01 µM/s
ν0	Basal calcium flux	0.0004 µM/s
ε	Calcium flux strength	5

k1	Maximal rate of calcium release	1.11 1/s
Ka	Calcium binding to activating site	0.08 µM
Kp	IP3 binding	0.13 µM
basal[1]	Basal IP3 production rate by PLC	0.3 µM/s
k1'[1]	Ligand-receptor association rate constant	4 1/(µM*s)
k2'[1]	Ligand-receptor dissociation rate constant	5 1/s
k3	Basal exchange rate of GTP for GDP on Gα subunit	0.01 1/s
k4	Rate constant for hydrolysis of GαGTP to GαGDP	2.5 1/s
k5	Encounter rate constant for ligand-receptor complex and G-protein	$2*10^{-5}$ 1/s
k6	Encounter rate constant for PLC and GαGTP	$2*10^{-5}$ 1/s
k7	Encounter rate constant for βγ and GαGDP	$1*10^{-5}$ 1/s
k8[2]	Rate constant for IP3 formation by PLC	700 1/s

[1] values not in the original model

[2] value averaged from original model value and literature values

iv. Initial Conditions for the Politi et al. model (with ligand/receptor/G-protein dynamics):

$[\mathrm{IP3}](0) = 0$; $[\mathrm{Ca}^{2+}](0) = 0.055\mu\mathrm{M}$; $[\mathrm{Ca}^{2+}(\mathrm{ER})](0) = 10.76\mu\mathrm{M}$; $[\mathrm{IP3r}](0) = 0$; $[\mathrm{LR}](0) = 0$; $[\mathrm{G\alpha GTP}] = 0$; [GαGDP] = 0; [PLC(active)] = 0

For the Politi et al. model, the parameter 'Vplc' represented the stimulant concentration 'C'. For the Politi et al. model that included ligand-receptor dynamics, 'L' represented 'C'.

C. Atri et al. model (Reference [8])

i. Model Description:

In the Atri et al. model, similar to the Politi et al. model, calcium modulates its release in a biphasic manner; further IP3 receptors (which mediate calcium release) are inactivated on a slower time scale than their activation. Collectively, these features result in calcium oscillations with periods on the order of seconds to 20 seconds (with constant IP3 levels). The model is able to reproduce several key features of calcium signaling in Xenopus laevis oocytes, despite only incorporating a single pool of calcium.

ii. Model equations:

(1)

$$\text{IP3rinf} = 1 - \frac{[\text{Ca}^{2+}]^2}{\text{k2}^2 + [\text{Ca}^{2+}]^2}$$

(2)

$$\text{Jch} = \text{kflux} * \mu * [\text{IP3r}] * \left(\text{b} + \frac{\text{V1} * [\text{Ca}^{2+}]}{\text{k1} + [\text{Ca}^{2+}]}\right)$$

(3)

$$\text{Jpump} = \frac{\gamma * [\text{Ca}^{2+}]}{\text{k}_\gamma + [\text{Ca}^{2+}]}$$

(4)

$$\text{Jleak} = \beta$$

(5)

$$\frac{\text{d}[\text{Ca}^{2+}]}{\text{dt}} = \text{Jch} - \text{Jpump} + \text{Jleak}$$

(6)

$$\frac{\text{d}[\text{IP3r}]}{\text{dt}} = \left(\frac{1}{\tau}\right) * (\text{IP3rinf} - [\text{IP3r}])$$

$[\text{Ca}^{2+}]$ = intracellular calcium; $[\text{IP3r}]$ = inositol triphosphate receptors not inactivated by calcium; IP3rinf = proportion of IP3 receptors not activated by calcium at steady state; Jch = calcium flux through the IP3 receptor; Jpump = calcium flux due to calcium-dependent pumping of calcium out of the cytosol; Jleak = calcium flux due calcium leaking into the cytosol

iii. Parameter Table:

Parameter	Description	Value
μ	Proportion of IP3 receptors that have their IP3 binding domain activated	Varied (unitless)
k1	Half-activation constant for IP3 receptor-mediated calcium	0.7 μM

	release	
γ	Maximum rate of calcium pumping	2 μM/s
b	Proportion of IP3 receptors that are activated in absence of calcium binding	0.111
V1	Proportion of IP3 receptors that are activated by calcium binding	0.889
β	Calcium leak rate	0.02 μM/s
τ	Time constant for IP3r inactivation	2 s
k_γ	Half-activation constant calcium pumping	0.1 μM
k2	Half-activation constant for IP3 receptor activation	0.7 μM
kflux	Maximum total calcium flux through all IP3 receptors	8.1 μM/s

iv. Initial Conditions:

$[Ca^{2+}]$ (0) = 0; [IP3r] (0) = 0

For the Atri et al. model, the parameter ‘μ’ represented the stimulant concentration ‘C’.

D. Li and Rinzel model (Reference [9])

i. Model Description:

The Li and Rinzel model represents a reduced version of the DeYoung and Keizer model [10], whereby elaborate IP3 receptor gating dynamics are greatly simplified based upon assumptions about the time scales of its activation and inactivation (similar to what was described for the Atri et al. model). The calcium oscillation periods produced this model are generally on the order of seconds, which is below what was observed experimentally. A version of the Li and Rinzel model that produced oscillations with periods on the order of hundreds of seconds was analyzed in the study by Sneyd et al. [3], and used here. In this version of the model, calcium feedback upon the IP3 receptor is omitted and calcium feedback upon IP3 metabolism is included.

ii. Model equations:

(1)

$$Jin = \propto 1 + \propto 2 * [IP3]$$

(2)

$$[Ca^{2+}(ER)] = \frac{[Ca^{2+}(total)] - [Ca^{2+}]}{\sigma}$$

(3)

$$h = \frac{Kd}{[Ca^{2+}] + Kd}$$

(4)

$$\frac{d[Ca^{2+}]}{dt} = \frac{fi}{Vi} * (\left(L + P * \left(\frac{[IP3] * [Ca^{2+}] * h}{([IP3] + Ki) * ([Ca^{2+}] + Ka)}\right)^3\right) * ([Ca^{2+}(ER)] - [Ca^{2+}]) - \left(\frac{Ve * [Ca^{2+}]^2}{Ke^2 + [Ca^{2+}]^2}\right) + \varepsilon * (Jin - \left(\frac{Vp * [Ca^{2+}]^2}{Kp^2 + [Ca^{2+}]^2}\right))$$

(5)

$$\frac{d[IP3]}{dt} = v4 * \left(\frac{[Ca^{2+}] + (1-\propto) * k4}{[Ca^{2+}] + k4}\right) - ir * [IP3]$$

(6)

$$\frac{d[Ca^{2+}(total)]}{dt} = \frac{fi}{Vi} * \varepsilon * (Jin - \left(\frac{Vp * [Ca^{2+}]^2}{Kp^2 + [Ca^{2+}]^2}\right))$$

$[Ca^{2+}]$ = intracellular calcium; [IP3] = inositol triphosphate; $[Ca^{2+}(total)]$ = total cellular calcium; h = IP3 receptors not inactivated by calcium; Jin = calcium flux into cytosol

iii. Parameter Table:

Parameter	Description	Value
v4	IP3 production rate constant	Varied (units: µM/s)
α	Calcium feedback strength on IP3 formation	0.97
k4	Half-activation constant for IP3 production	1.1 µM
ir	IP3 degradation rate constant	0.08 1/s
α1	Basal calcium influx	400 µM/s
α2	Calcium influx activation rate constant	100 1/s

ε	Calcium flux strength at plasma membrane	0.01
fi	Calcium flux scaling factor	0.01
Vi	Calcium flux scaling factor	4
Vp	Maximum rate of calcium release from PMCA pump	2000 µM/s
L	Calcium leak rate constant	0.37 1/s
P	Calcium release rate constant from IP3 receptors	26640 1/s
Ki	Calcium binding to inhibiting site	1 µM
Ka	Calcium binding to activating site	0.4 µM
Ve	Maximum rate of calcium release from SERCA pump	400 µM/s
Ke	Half-activation constant for SERCA pump	0.2 µM
Kd	Half-activation constant for activate IP3 receptors	0.4 µM
σ	Ratio of effective volumes of the ER/cytosol	0.185
Kp	Half-activation constant for PMCA pump	0.3 µM

iv. Initial Conditions:

$[Ca^{2+}]$ (0) = 0.15 µM; [IP3] (0) = 0; $[Ca^{2+}(total)]$(0) = 72.178 µM

For the Li and Rinzel model, the parameter 'v4' was used to represent the stimulant concentration 'C'.

E. Dupont et al. model (Reference [11])

i. Model description:

The Dupont et al. model is based upon experimental observations that IP3 metabolism, specifically from IP3-3 kinase and IP3-5 phosphatase, significantly affect calcium signaling dynamics. In this model, external stimulation leads to IP3 release, which then results in calcium release. Calcium then engages the two aforementioned enzymes, which result in reduced IP3 levels. This feedback mechanism results in calcium oscillations, and is able to reproduce several experimentally observed calcium signaling behaviors.

ii. Model equations:

(1)

$$k^{+} = \frac{k^{-}}{\left(k_{in}^{h}\right)^{4}}$$

(2)

$$\frac{d[R]}{dt} = (k^{+} * [Ca^{2+}]^{4}) * \left(\frac{1 - [R]}{1 + \left(\frac{[Ca^{2+}]}{kact}\right)^{3}}\right) - k^{-} * [R]$$

(3)

$$\frac{d[Ca^{2+}]}{dt} = k1 * (b + Ira) * \left([Ca^{2+}(total)] - [Ca^{2+}] * (\propto +1)\right) - Vmp * \frac{[Ca^{2+}]^{np}}{Kp^{np} + [Ca^{2+}]^{np}}$$

(4)

$$Irable = (1 - [R]) * \frac{[IP3]}{kip + [IP3]}$$

(5)

$$Ira = Irable * \frac{1}{1 + \left(\frac{kact}{[Ca^{2+}]}\right)^{3}}$$

(6)

$$\frac{d[IP3]}{dt} = vplc - v3k - v5p$$

(7)

$$vplc = \gamma * Vplc$$

(8)

$$v3k = Vk * \frac{[IP3]}{Kk + [IP3]} * \frac{[Ca^{2+}]^{nd}}{Kd^{nd} + [Ca^{2+}]^{nd}}$$

(9)

$$v5p = Vp1 * \frac{[IP3]}{Kp1 * \left(1 + \left(\frac{[IP4]}{Kp2}\right)\right) + [IP3]}$$

(10)

$$\frac{d[IP4]}{dt} = v3k - v5p' - k * [IP4]$$

(11)

$$v5p' = Vp2 * \frac{[IP4]}{Kp2 * \left(1 + \left(\frac{[IP3]}{Kp1}\right)\right) + [IP4]}$$

$[Ca^{2+}]$ = intracellular calcium; $[R]$ = fraction of desensitized IP3 receptor; $[IP3]$ = inositol triphosphate; [IP4] = inositol tetrakisphosphate; Irable = fraction of activable IP3 receptors; Ira = fraction of activated IP3 receptors; vplc = rate of IP3 metabolism by PLC; v3k = rate of IP3 metabolism by IP3 3-kinase; v5p = rate of IP3 metabolism by IP3 5-phosphatase; v5p' = rate of IP4 metabolism by IP4 5-phosphatase; k^{+} = association rate constant of calcium for the IP3 receptor

iii. Parameter Table:

Parameter	Description	Value
γ	Level of external stimulation	Varied (unitless)
kact	Calcium binding to activating site	0.56 µM
k_{in}^{h}	Calcium binding to inhibiting site	0.15 µM
k1	Maximum total calcium flux through all IP3 receptors	2.57 1/s
k^{-}	IP3 receptor dissociation rate constant	0.5 1/s
Vplc	Maximal velocity of IP3 synthesis	1.3 µM/s
Vk	Maximal velocity of IP3 3-kinase	0.5 µM/s
Kk	Michaelis constant of IP3 3-kinase	1 µM
Kd	Threshold for IP3 3-kinase activation by calcium	0.3 µM
nd	Hill coefficient for IP3 3-kinase activation by calcium	2
Vp1	Maximal velocity of 5-phosphatase (IP3 substrate)	5 µM/s
Kp1	Michaelis constant of 5-phosphatase (IP3 substrate)	10 µM

Vp2	Maximal velocity of 5-phosphatase (IP4 substrate)	0.2 µM/s
Kp2	Michaelis constant of 5-phosphatase (IP4 substrate)	2 µM
k	Rate constant of linear degradation of IP4	0.01 1/s
ni	Hill coefficient for inactivation of the IP3 receptor by calcium	4
na	Hill coefficient for activation of the IP3 receptor by calcium	3
k1	Calcium transfer rate between intracellular stores and cytosol	2.57 µM/s
α	Ratio of the volumes of the intracellular stores and the cytosol	0.1
Vmp	Maximal velocity of calcium pumping	4 µM/s
Kp	Half-activation constant for calcium pumping	0.35 µM
np	Hill coefficient for calcium pumping	2
Kip	Dissociation constant between IP3 and IP3 receptor	1 µM
$[\mathrm{Ca}^{2+}\ (\mathrm{total})]$	Total cellular calcium	80 µM

iv. Initial Conditions:

[R] (0) = 0 µM; $[\mathrm{Ca}^{2+}]$ (0) = 0 µM; [IP3](0) = 0; [IP4](0) = 0

For the Dupont et al. model, the parameter 'γ' was used to represent the stimulant concentration 'C'.

F. Kummer et al. model (Reference [12])

i. Model description:

The Kummer et al. model considers G-protein and PLC dynamics in the context of generation of calcium oscillations. In particular, the model consists of negative feedback by calcium and PLC on receptor-mediated G-protein activation. In addition, the nature of G-protein activation can be modulated by changing receptor relevant parameters. The model is able to display regular calcium oscillation patterns as well as chaotic calcium bursting upon constant stimulation.

ii. Model equations:

$$\frac{d[\mathrm{G} \propto \mathrm{GTP}]}{dt} = \mathrm{k1} + \mathrm{k2} * [\mathrm{G} \propto \mathrm{GTP}] - \frac{\mathrm{k3} * [\mathrm{G} \propto \mathrm{GTP}] * [\mathrm{PLC}]}{1 + \mathrm{K4}} - \frac{\mathrm{k5} * [\mathrm{G} \propto \mathrm{GTP}] * [\mathrm{Ca}^{2+}]}{[\mathrm{G} \propto \mathrm{GTP}] + \mathrm{K6}}$$

$$\frac{d[PLC]}{dt} = k7 * [G \propto GTP] - \frac{k8 * [PLC]}{[PLC] + K9}$$

$$\frac{d[Ca^{2+}]}{dt} = k10 * [Ca^{2+}] * [PLC] * \frac{[Ca^{2+}(ER)]}{[Ca^{2+}(ER)] + K11} + k12 * [PLC] + k13 * [G \propto GTP] - k14 * \frac{[Ca^{2+}]}{[Ca^{2+}] + K15} - k16 * \frac{[Ca^{2+}]}{[Ca^{2+}] + K17}$$

$$\frac{d[Ca^{2+}(ER)]}{dt} = -k10 * [Ca^{2+}] * [PLC] * \frac{[Ca^{2+}(ER)]}{[Ca^{2+}(ER)] + K11} + k16 * \frac{[Ca^{2+}]}{[Ca^{2+}] + K17}$$

$[G \propto GTP]$ = activated G-protein; $[PLC]$ = phospholipase C; $[Ca^{2+}]$ = intracellular calcium; $[Ca^{2+}(ER)]$ = endoplasmic reticulum calcium

iii. Parameter Table:

Parameter	Description	Value (all unitless)
k2	G-protein activation rate constant	Varied
k1	Spontaneous G-protein activation rate	0.09
k3	Maximum PLC-mediated G-protein inactivation rate constant	0.64
K4	Half-activation constant for PLC-mediated G-protein inactivation	0.19
k5	Maximum calcium-mediated G-protein inactivation rate constant	4.88
K6	Half-activation constant for calcium-mediated G-protein inactivation	1.18
k7	G-protein-mediated PLC activation rate constant	2.08
k8	Maximum PLC inactivation rate constant	32.24
K9	Half-activation constant for PLC inactivation	29.09
k10	Maximum IP3 production rate constant	5
K11	Half-activation constant for IP3 production	2.67
k12	IP3-mediated calcium influx	0.7
k13	G-protein-mediated calcium influx	13.58

k14	Maximum rate of calcium release from SERCA pump	153
K15	Half-activation constant for SERCA pump	0.16
k16	Maximum rate of calcium release from PMCA pump	4.85
K17	Half-activation constant for PMCA pump	0.05

iv. Initial Conditions for the Kummer et al. model (all unitless):

$[G \propto GTP]$ (0) = 0.01; $[PLC]$ (0) = 0.01; $[Ca^{2+}]$ (0) = 0.01; $[Ca^{2+}(ER)]$ (0) = 20

For the Kummer et al. model, the parameter 'k2' was used to represent the stimulant concentration 'C'.

G. Cuthbertson and Chay model (Reference [13])

i. Model Description: Mechanistically the Cuthbertson and Chay model is very similar to the Chay et al. model described previously.

ii. Model equations:

(1)

$$\frac{d[G \propto GTP]}{dt} = kg * (G0 - [G \propto GTP] - 4 * [PLC]) - 4 * kp * [G \propto GTP]^4 * (P0 - PLC) - hg * [G \propto GTP]$$

(2)

$$\frac{d[IP3]}{dt} = kd * [PLC] - hd * [IP3] + ld$$

(3)

$$\frac{d[Ca^{2+}]}{dt} = \rho kc * (\frac{[IP3]^3}{Ks^3 + [IP3]^3}) - \rho * [Ca^{2+}] + \rho lc$$

(4)

$$\frac{d[PLC]}{dt} = kp * [G \propto GTP]^4 * (P0 - PLC) - hp * [PLC]$$

(5)

$$kn = knprime * \left(\frac{[IP3]^2}{Kd^2 + [IP3]^2}\right); \quad \text{where } kn = kp, hp, kd$$

$[G \propto GTP]$ = activated G-protein; $[IP3]$ = inositol triphosphate; $[Ca^{2+}]$ = intracellular calcium; $[PLC]$ = phospholipase C

iii. Parameter Table:

Parameter	Description	Value
kg	G-protein activation rate constant (activated receptor contribution)	Varied (units: 1/s)
hg	G-protein hydrolysis rate constant	0 (units: 1/s)
hd	IP3 hydrolysis rate constant	100 1/s
ld	IP3 leak rate	250 nM/s
ρkc	Calcium activation rate by IP3	$9*10^4$ nM/s
ρhc	Calcium removal rate constant	1 1/s
ρlc	Calcium leak rate	200 nM/s
Ks	Half-activation constant for IP3	300 nM
Kd	Half-activation constant for DAG	25 nM
basal	Basal G-protein activation rate	0.005 1/s
G0	Total G-protein Concentration	200 nM
P0	Total PLC Concentration	10 nM
kdprime	DAG-mediated PLC activation rate constant	700 1/s
kpprime	DAG-mediated G-protein/PLC dissociation rate constant	$2*10^{-7}$ 1/(nM^4*s)
hpprime	DAG-mediated PLC deactivation rate constant	0.5 1/s

iv. Initial Conditions:

$[G \propto GTP](0) = 0$; $[IP3](0) = 0$; $[Ca^{2+}](0) = 200$ nM; $[PLC](0) = 0$

For the Cuthbertson and Chay model, the parameter ‘kg’ was used to represent the stimulant concentration ‘C’.

H. Tyson et al. model (Circadian model- Reference [4])

i. Rate equations for the Tyson et al. model (Circadian model):

(1)

$$q = \frac{2}{1 + (1 + 8 * \text{Keq} * [\text{Protein}])^{0.5}}$$

(2)

$$\frac{d[\text{mRNA}]}{dt} = \frac{\text{vm}}{1 + \left([\text{Protein}] * \frac{1 - q}{2 * \text{Pcrit}}\right)^2} - \text{km} * [\text{mRNA}]$$

(3)

$$\frac{d[\text{Protein}]}{dt} = \text{vp} * [\text{mRNA}] - \left(\frac{\text{kp1} * [\text{Protein}] * q + \text{kp2} * [\text{Protein}]}{\text{Jp} + [\text{Protein}]}\right) - \text{kp3} * [\text{Protein}]$$

[mRNA] = PER/TIM mRNA; [Protein] = PER/TIM protein; q = fraction of protein monomer

ii. Parameter table:

Parameter	Description	Value*
vm	Maximum mRNA synthesis rate	Varied (Cm/hr)
km	First-order rate constant for mRNA degradation	0.1 1/hr
vp	Rate constant for translation of mRNA	0.5 Cp/(Cm*hr)
kp1	Vmax for monomer phosphorylation	10 Cp/hr
kp2	Vmax for dimer phosphorylation	0.03 Cp/hr
kp3	First-order rate constant for proteolysis	0.1 1/hr
Keq	Equilibrium constant for dimerization	200 1/Cp
Pcrit	Dimer concentration at the half-maximum transcription rate	0.1 Cp
Jp	Michaelis constant for protein kinase (DBT)	0.05 Cp

* Cm and Cp represent characteristic concentrations for mRNA and protein respectively (as presented in the original publication)

iii. Initial Conditions:

[mRNA](0) = 0; [Protein](0) = 0

For the Tyson et al. model, the parameter 'vm' was used to represent the stimulant 'C'.

I. Goldbeter model (Circadian model- Reference [5])

i. Model equations:

(1)

$$\frac{dM}{dt} = vs * \left(\frac{Ki^4}{Ki^4 + Pn^4}\right) - vm * (\frac{M}{Km + M})$$

(2)

$$\frac{dP0}{dt} = ks * M - v1 * \left(\frac{P0}{K1 + P0}\right) + v2 * (\frac{P1}{K2 + P1})$$

(3)

$$\frac{dP1}{dt} = v1 * \left(\frac{P0}{K1 + P0}\right) - v2 * \left(\frac{P1}{K2 + P1}\right) - v3 * \left(\frac{P1}{K3 + P1}\right) + v4 * \left(\frac{P2}{K4 + P2}\right)$$

(4)

$$\frac{dP2}{dt} = v3 * \left(\frac{P1}{K3 + P1}\right) - v4 * \left(\frac{P2}{K4 + P2}\right) - k1 * P2 + k2 * Pn - vd * \left(\frac{P2}{Kd + P2}\right)$$

(5)

$$\frac{dPn}{dt} = k1 * P2 - k2 * Pn$$

M = PER mRNA concentration; P0 = unphosphorylated PER protein concentration; P1 = monophosphorylated PER protein concentration; P2 = bisphosphorylated PER protein concentration; Pn = nuclear bisphosphorylated PER protein concentration

ii. Parameter Table:

Parameter	Description	Value
vs	Maximum mRNA cytosolic transport rate	Varied (units: µM/hr)
vm	Maximum mRNA degradation rate	0.65 µM/hr
Km	Half-activation of mRNA degradation	0.5 µM

ks	mRNA translation rate constant	0.38 1/hr
vd	Maximum bisphosphorylated PER protein degradation rate	0.95 µM/hr
k1	Bisphosphorylated PER protein nuclear transport rate constant	1.9 1/hr
k2	Nuclear bisphosphorylated PER protein concentration degradation rate constant	1.3 1/hr
Ki	Half-activation of nuclear bisphosphorylated PER protein-induced mRNA production	1 µM
Kd	Half-activation of bisphosphorylated PER protein degradation	0.2 µM
K1	Half-activation of kinase activity for unphosphorylated PER protein	2 µM
K2	Half-activation of phosphatase activity for monophosphorylated PER protein	2 µM
K3	Half-activation of kinase activity for monophosphorylated PER protein	2 µM
K4	Half-activation of phosphatase activity for bisphosphorylated PER protein	2 µM
v1	Maximum kinase activity rate for unphosphorylated PER protein	3.2 µM/hr
v2	Maximum phosphatase activity rate for monophosphorylated PER protein	1.58 µM/hr
v3	Maximum kinase activity rate for monophosphorylated PER protein	5 µM/hr
v4	Maximum phosphatase activity rate for bisphosphorylated PER protein	2.5 µM/hr

iii. Initial Conditions:

M(0) = 1 µM; P0(0) = 0; P1(0) = 0, P2(0) = 0; Pn(0) = 0

For the Goldbeter Model, the parameter ‘vs’ was used to represent the stimulant concentration ‘C’.

References:

1. Schreiber G, Henis YI, Sokolovsky M (1985) Rate constants of agonist binding to muscarinic receptors in rat-brain medulla - evaluation by competition kinetics. J Biol Chem 260: 8795-8802.
2. Mahama PA, Linderman JJ (1994) Calcium Signaling in Individual Bc(3)H1 Cells - Speed of Calcium Mobilization and Heterogeneity. Biotechnol Prog 10: 45-54.
3. Sneyd J, Tsaneva-Atanasova K, Reznikov V, Bai Y, Sanderson MJ, et al. (2006) A method for determining the dependence of calcium oscillations on inositol trisphosphate oscillations. Proc Natl Acad Sci USA 103: 1675-1680.
4. Tyson JJ, Hong CI, Thron CD, Novak B (1999) A simple model of circadian rhythms based on dimerization and proteolysis of PER and TIM. Biophys J 77: 2411-2417.
5. Goldbeter A (1995) A Model for Circadian Oscillations in the Drosophila Period Protein (Per). Proc R Soc Lond Ser B- Biol Sci 261: 319-324.
6. Chay TR, Lee YS, Fan YS (1995) Appearance of phase-locked Wenckebach-like rhythms, devil's staircase and unviersality in intracellular calcium spikes in nonexcitable cell models. J Theor Biol 174: 21-44.
7. Politi A, Gaspers LD, Thomas AP, Hofer T (2006) Models of IP3 and Ca2+ oscillations: Frequency encoding and identification of underlying feedbacks. Biophys J 90: 3120-3133.
8. Atri A, Amundson J, Clapham D, Sneyd J (1993) A Single-Pool Model for Intracellular Calcium Oscillations and Waves in the Xenopus-Laevis Oocyte. Biophys J 65: 1727-1739.
9. Li YX, Rinzel J (1994) Equations for INSP(3) Receptor-mediated [Ca2+](i) oscillations derived from a detailed kinetic-model- a Hodgkin-Huxley-like formalism. J Theor Biol 166: 461-473.
10. Deyoung G, Keizer J (1993) A Single Pool Inositol 1,4,5-Triphosphate Receptor Based Model for Agonist Stimulated Calcium Oscillations. Biophys J 64: A229-A229.
11. Dupont G, Erneux C (1997) Simulations of the effects of inositol 1,4,5-trisphosphate 3-kinase and 5-phosphatase activities on Ca2+ oscillations. Cell Calcium 22: 321-331.
12. Kummer U, Olsen LF, Dixon CJ, Green AK, Bornberg-Bauer E, et al. (2000) Switching from simple to complex oscillations in calcium signaling. Biophys J 79: 1188-1195.
13. Cuthbertson KSR, Chay TR (1991) Modeling receptor-controlled intracellular calcium oscillators. Cell Calcium 12: 97-109.
